# Increased water use efficiency leads to decreased precipitation sensitivity of tree growth, but is offset by high temperatures

**DOI:** 10.1007/s00442-021-04892-0

**Published:** 2021-03-20

**Authors:** Kelly A. Heilman, Valerie M. Trouet, Soumaya Belmecheri, Neil Pederson, Melissa A. Berke, Jason S. McLachlan

**Affiliations:** 1 Department of Biological Sciences, University of Notre Dame, Notre Dame, IN USA; 2 Laboratory of Tree-Ring Research, University of Arizona, Tucson, AZ USA; 3 Harvard Forest, Harvard University, Petersham, MA USA; 4 Department of Civil and Environmental Engineering, University of Notre Dame, Notre Dame, IN USA

**Keywords:** Water Use Efficiency, Tree growth, Climate sensitivity, CO2, *δ*^13^*C*

## Abstract

Both increases in temperature and changes in precipitation may limit future tree growth, but rising atmospheric CO_2_ could offset some of these stressors through increased plant Water Use Efficiency (WUE). The net balance between the negative impacts of climate change and positive effects of CO_2_ on tree growth is crucial for ecotones, where increased climate stress could drive mortality and shifts in range. Here, we quantify the effects of climate, stand structure, and rising CO_2_ on both annual tree-ring growth increment and intrinsic WUE (iWUE) at a savanna-forest boundary in the Upper Midwest United States. Taking a Bayesian hierarchical modelling approach, we find that plant iWUE increased by ~ 16–23% over the course of the twentieth century, but on average, tree-ring growth increments do not significantly increase. Consistent with higher iWUE under increased CO_2_ and recent wetting, we observe a decrease in sensitivity of tree growth to annual precipitation, leading to ~ 35–41% higher growth under dry conditions compared to trees of similar size in the past. However, an emerging interaction between summer maximum temperatures and annual precipitation diminishes the water-savings benefit under hot and dry conditions. This decrease in precipitation sensitivity, and the interaction between temperature and precipitation are strongest in open canopy microclimates, suggesting that stand structure may modulate response to future changes. Overall, while higher iWUE may provide some water savings benefits to growth under normal drought conditions, near-term future temperature increases combined with drought events could drive growth declines of about 50%.

**Supplementary Information:**

The online version contains supplementary material available at 10.1007/s00442-021-04892-0.

## Introduction

Future increases in temperature, changes in precipitation regimes, and elevated atmospheric CO_2_ have the potential to drive large shifts in tree growth and distribution (Allen and Breshears 1998; Scheller and Mladenoff 2008; Reinmann and Hutyra 2017; Nolan et al. 2018). Forecasting these responses is difficult, as concurrent changes can have contrasting effects on vegetation, leading to uncertainty over which systems will benefit, and which will be disadvantaged by global change. The net effects of divergent environmental changes on trees are particularly critical at ecotone or biome boundaries, where drought stress, amplified by microclimatic feedbacks could drive severe tree growth declines and a shifts in distribution (Breshears et al. 2005; Adams et al. 2009; Allen et al. 2015; Clark et al. 2016; Charney et al. 2016; Druckenbrod et al. 2019). In contrast to climate changes, the benefits of increased atmospheric CO_2_ could alleviate some drought stress, and lead to enhanced tree growth and biomass (Ainsworth and Long 2005; Norby and Zak 2011). Quantifying long term responses of tree growth to joint changes in temperature, precipitation, and CO_2_ is critical to forecast future function at biome boundaries.

Enhanced CO_2_ increases plant Water Use Efficiency (WUE) and generally stimulates plant biomass at ecosystem scales, but the magnitude and persistence of this effect depends on other limiting factors to plant growth, such as nutrients or climate (Ainsworth and Long 2005; Norby and Zak 2011; Keenan et al. 2013). Indeed, many global change researchers seek to answer the question “What will determine the winners and losers in the competition to capitalize on the sudden increased availability of atmospheric CO_2_?” (Monson 2003). Tree-ring stable *δ*^13^*C* isotopes also document a ~ 10–30% increase in intrinsic WUE (iWUE) over the last century, but paradoxically, the requisite increases in tree-ring growth are rarely detected (Peñuelas et al. 2011; Andreu‐Hayles et al. 2011; Silva and Anand 2013; Tognetti et al. 2014; van der Sleen et al. 2015; Frank et al. 2015; Fernández‐de‐Uña et al. 2016; Levesque et al. 2017; Hararuk et al. 2019). The reasons for this paradox are widely debated, highlighting a need to understand how other limiting growth factors, such as climate change (Wyckoff and Bowers 2010; Granda et al. 2014), interannual variations in climate (McCarroll and Loader 2004), and stand competition (Fernández‐de‐Uña et al. 2016), modify the long term effects of increased CO_2._

Climate and CO_2_ changes can have potentially competing effects on tree growth that are especially critical in temperate savanna-forest ecotones. Increased frequency and intensity of high temperature drought events (Cook et al. 2015) could drive reduced radial tree growth (Cook et al. 2015; Clark et al. 2016; Charney et al. 2016), but rising atmospheric CO_2_ may reduce stress of drought events by increasing WUE. Indeed, recent evidence points to reduced tree drought sensitivity in temperate eastern forests and savannas of the US (Wyckoff and Bowers 2010; Maxwell et al. 2016), a shift that is consistent with both increased wetting and relative humidity (Stahle et al. 2020) and with higher iWUE. However, it remains to be seen to what degree rising CO_2_ alleviates negative impacts of climate change in this region.

Stand structural differences that are often present at ecotones could either amplify or diminish the potential benefits of increasing WUE, as well as the impacts of climate change (Chen et al. 1993; Frey et al. 2016; Reinmann and Hutyra 2017). For example, competition in closed forests may lead to higher climate sensitivity (Fernández-de-Uña et al. 2015), and a stronger response to increased WUE. Hotter microclimates in open canopy systems (Chen et al. 1993; Frey et al. 2016), can drive plant stress, resulting in large growth declines under hotter droughts (Gea-Izquierdo et al. 2009; Fernández‐de‐Uña et al. 2016; Reinmann and Hutyra 2017), and can drive physiological responses to light and heat stress (Litvak et al. 1996; Monson et al. 2016). Growth declines that lead to increased mortality would have feedbacks that further open the canopy (Allen and Breshears 1998; Breshears et al. 2005), while forest microclimates buffer trees from temperature stress and mortality (Adams et al. 2009), maintaining the closed canopy. Thus, understanding how stand structure differences can modulate CO_2_ and climate effects could inform forecasts of vegetation across temperate forest-savanna ecotones, as well as biome boundaries with differing stand structures across the globe.

**Fig. 1:**
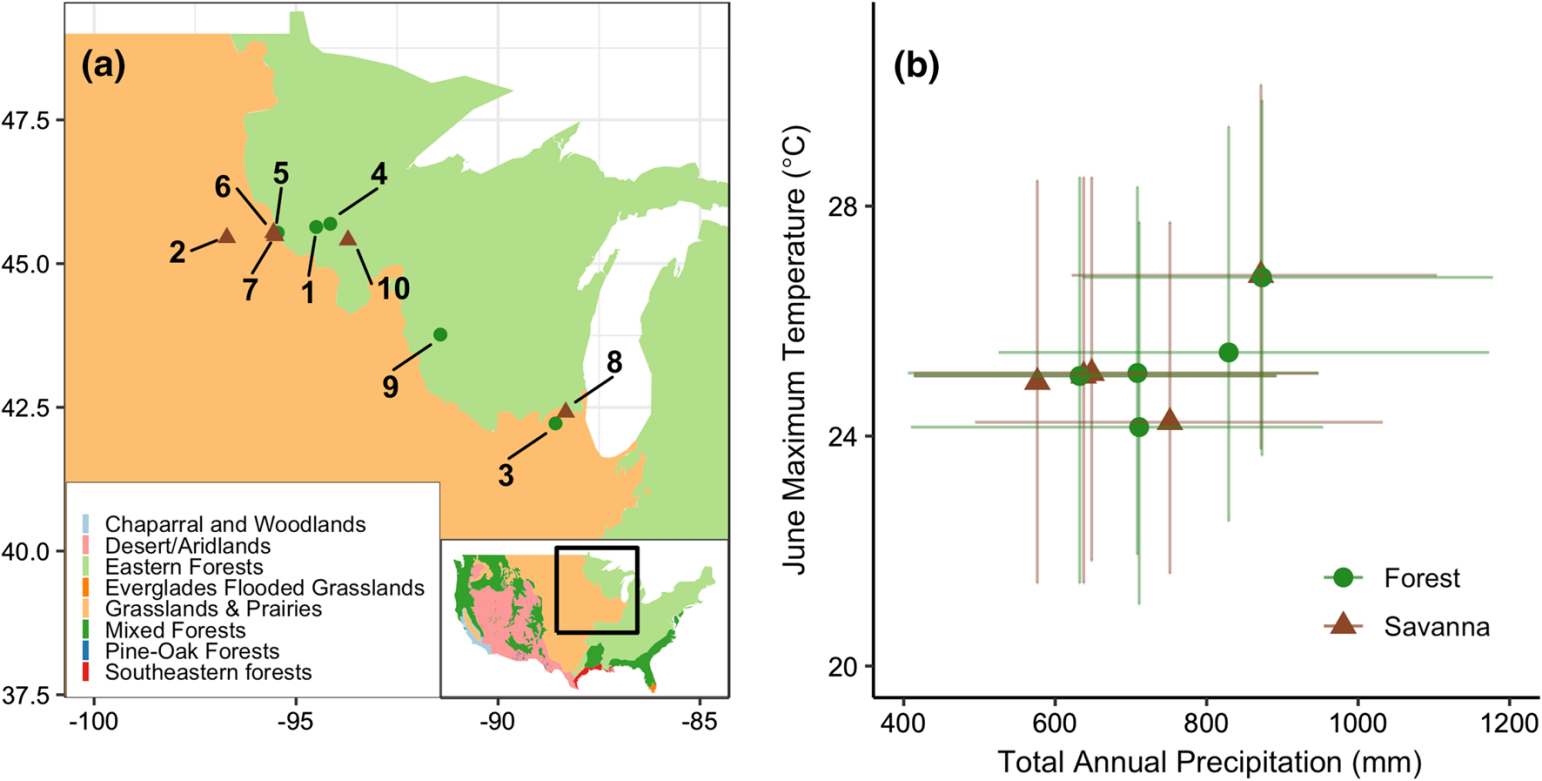
Distribution of nine savanna and forest sites sampled across geographic and climate space. **a** Map of sites in the upper midwestern United States. Background includes biome designations (from Olson et al., 2001). **b** Average water year precipitation and the average June maximum temperatures for each site from 1895 to 2014 (see methods). Error bars represent 95% quantiles. Green circles and brown triangles indicate forests and savannas, respectively

Here, we quantify the joint impacts of climate and CO_2_ on growth and iWUE using tree-ring growth increments and *δ*^13^*C* from nine sites across a savanna-forest ecotone boundary in North America (Fig. 1a). Using a Bayesian hierarchical framework, we model both tree growth and intrinsic WUE of *Quercus spp.* trees of two different cohorts: those that experienced either low CO_2_ (established before 1895) or high CO_2_ levels (established after 1895, but before 1950), that are similar in most other aspects (i.e., age, tree size, stand structure). We address several key questions: 1). Does iWUE increase with higher CO_2_ levels? 2). If so, does increased iWUE result in a detectable increase in radial tree growth? 3). Does higher iWUE decrease growth sensitivity to precipitation? 4). Do concurrent changes in temperature have a positive or negative effect on tree growth? 5). How does stand structure mediate the joint responses to climate and CO_2_?

We hypothesize that elevated CO_2_ increases tree iWUE. If precipitation is a primary limiting factor to tree growth, then higher iWUE will provide a water-savings benefit that results in both lower sensitivity to precipitation and an increase in overall growth consistent with CO_2_ fertilization. However, if high temperatures limit tree growth and outweigh the effects of increased iWUE, then we expect no change or growth declines. Finally, we expect that different stand structure microclimates will modulate growth responses such that savanna trees are more temperature sensitive because of their exposed crowns, but forest trees are more precipitation sensitive due to higher tree competition for water. In summary, increasing WUE over time should decrease precipitation sensitivity of growth, but higher temperature should have an increasingly negative effect on growth that is more apparent in savanna systems.

## Methods

### Study region

Our study region spans the savanna-forest boundary in the Upper Midwest of North America (Fig. 1a). A sharp boundary between oak savannas and forests was a major feature of the region in the 1800s (Goring et al. 2016), but today agriculture and forests dominate, though some small savannas persist. Given this history, future droughts could shift forests to open systems (Scheller and Mladenoff 2008; Frelich and Reich 2010), but increased iWUE might reduce drought stress (Wyckoff and Bowers 2010). As such, this is an ideal study system for understanding how the competing effects of environmental change play out at across an ecotone.

We selected a total of nine sites (five savannas and four closed forests) situated along an increasing moisture gradient from West to East (Fig. 1a), spanning mean annual precipitations from 577 to 873 mm (95% CI 372–178 mm) (Fig. 1b, Table S1). Sites also vary along a North to South temperature gradient spanning ~ 24.1–26.8 °C (95% CI 21–30 °C) (Fig. 1b, Table S2).

We sampled 1–3 cores from trees > 3 cm DBH, and censused all trees within a single 15-m radius plot at each site. This dendroecological sampling minimizes biases associated with tree size or age common in tree-ring collections for climatic reconstructions, and captures the range of growth-climate responses (Davis et al. 2009; Brienen et al. 2012; Babst et al. 2014; Dye et al. 2016; Klesse et al. 2018). We focus on *Quercus spp.*, as it is one of the only taxa present in both forest types.

### Tree-ring growth measurements and crossdating

Cores were dried, mounted with hide glue, sanded, and visually crossdated. We measured ring widths to the nearest micrometer using a Velmex measuring station and Tellervo Software (Brewer 2014). Crossdating of whole-wood ring widths were statistically tested and verified using COFECHA, and site summary statistics calculated using DPLR (Bunn 2010) (Table S1).

### Tree-ring growth hierarchical sampling design

The effects of CO_2_ may diminish with tree age, and trends in atmospheric CO_2_ concentration covary with ontogenetic trends in tree growth (Voelker et al. 2006; Brienen et al. 2017b; Hararuk et al. 2019). To account for these biases, we developed a novel data analysis design that separates trees into two cohort groups and two stand structure groups (Table S3). The “Past” cohort consists of trees established before 1895 (date with earliest available climate data), under low CO_2_ conditions (294–310 ppm). The “Modern” cohort consists of trees established between 1895 and 1950 and experienced higher CO_2_ conditions (310–400 ppm). With this grouping, past and modern trees had means of 36 and 27 years, respectively. Subsampling of the dataset yields some sites with small within-cohort sample sizes (Table S2). To address this, we compare growth of trees in the modern cohort during the modern time period (1950–2015, *n* = 49 trees) to the past cohort during the past time period (1895–1949, *n* = 54 trees), and compare savannas (*n* = 72 trees) and closed forests (*n* = 31 trees) (Table S2).

### Climatic data and preliminary tree ring analysis

Climate data for each site are compiled from the 1895–1980 and the 1981–2014 4 km PRISM data products (PRISM Climate Group, Oregon State University 2004). As there are no stand level records of climate, and our stands are located in relatively low elevation, and flat terrain, extractions of gridded PRISM data products should adequately represent our sites. Preliminary static and moving correlations of ring widths and all monthly temperature, vapor pressure deficit, and precipitation climate parameters were used to select climatic variables included the tree-ring growth model. Tree-ring growth is strongly positively correlated with water year precipitation (October–September) and negatively correlated to June maximum temperature (Fig. S1).

Consistent with observed drought sensitivity changes in the region (Wyckoff and Bowers 2010; Maxwell et al. 2016), moving correlation analysis of detrended site chronologies indicates shifting climate sensitivity over time (Fig. S2). Regression slopes of spline detrended tree-ring time series and precipitation also have temporal changes (Figs. S3-4). However, these comparisons omit the hierarchical structure of our dataset, have reduced sample sizes for some sites, and omit other factors affecting growth. We address these issues with a novel Bayesian model, which leverages hierarchies in our data, borrows strength across cohorts, and assesses multiple drivers of growth (Hobbs and Hooten 2015; Dietze 2017).

### Bayesian hierarchical model of tree growth

Our hierarchical approach is rooted in the linear aggregate tree growth model, which identifies climate, tree age, size, and disturbances as the dominant predictors of annual tree-ring growth (Cook 1990). Similar to previous models of tree-ring growth (Cook 1990; Ogle et al. 2015), we include lagged effects of the previous 1–2 years of growth and DBH. In doing this, we account for trends in tree size and age that statistical detrending methods target (Voelker et al. 2006; Bowman et al. 2013; Foster et al. 2016). We chose raw tree ring widths as our response variable for three reasons: 1. Absolute tree growth is relevant to carbon uptake, 2. Statistical detrending steps can remove the long term trends of interest and introduce biases (Freckleton 2002), and 3. Basal Area Increment still contains some size trends (Bowman et al. 2013).

We include two climate variables with strong correlations with growth: total water year precipitation and June maximum temperature (Figure S1). We use these climate covariates and their interaction rather than an integrated moisture index (SPEI, PDSI, VPD, etc.), as this allows us to parse temperature and precipitation effects. Finally, some climate shifts occurred over our climate record (Table S2), which might impact the estimated drought responses (Maxwell et al. 2016; Helcoski et al. 2019). To reduce the influence of long term trends in moisture on climate responses, we ran our analysis using only the years of strong drought across the time period (based on the top 75% quantiles of June–August meteorological PDSI for each site and ageclass). This results in a total of 1503 records of annual tree growth, split into 1124 years of training data and 379 years of testing data. We use these results in the main text, but also ran the growth model with all years, to similar results (Fig. S5). We assessed model fit and model selection based on the *R*^2^ of predicted tree growth versus held-out observations, Mean Squared Prediction Error (MSPE), model bias, and Deviance Information Criterion (DIC) (Table S3).

Each annual observation (i) of log-transformed radial tree growth for each tree (t) is modeled as a function of $g\left(\alpha , \beta , Precip, DBH, MaxTemp, prev(-1\right), prev\left(-2\right))$, where *Precip* is scaled total water year precipitation for the current growth year, *DBH* is scaled tree size, *MaxTemp* is the scaled June maximum temperatures associated with each observation (i) and tree (t). The climate and DBH covariates are scaled by the mean and standard deviation of their respective cohort and structure classes. We also explored an interaction between *MaxTemp* and *Precip* (*MaxTempxPrecip*) (Table S3). The previous 1 and 2 years of tree growth are *lag(-1)* and *lag(-2)* parameters*.* Random effects for cohort groups *c* on all the $\beta$ parameters estimate parameter variation across cohorts and structures. Since site specific conditions might affect average tree growth, we include a random intercept for each site (s):

1 $${g= \alpha }_{s}+{\beta }_{1c}\times {Precip}_{it}+{\beta }_{2c}\times {MaxTemp}_{it}+{\beta }_{3c}\times {MaxTempxPrecip}_{it}+ {\beta }_{4c}\times {\mathrm{log}(growth-1)}_{it} +{\beta }_{5c}\times {\mathrm{log}\left(growth-1\right)}_{it}+{\beta }_{6c}\times {DBH}_{it}$$

Thus, we estimate the tree growth as a function of *g()* and process uncertainty (${\sigma }_{p}^{2})$:

2 $${\mathrm{log}(TrueGrowth)}_{isc} \sim normal(g, {\sigma }_{p}^{2})$$

Random effects allow parameter sensitivity to vary across cohort (c) while borrowing statistical strength across our dataset. We fit this model twice to estimate parameter sensitivity across cohorts and stand structure types. For the first model fit (cohort-only model), cohort (c) classes are Modern and Past. For the second (structure–cohort model), cohorts (c) represent the four combinations of age class and stand structure: “Modern–Savanna”, “Modern–Forest”, “Past–Savanna”, and “Past–Forest”. The advantage of this approach is that the $\beta$ random effects group all the trees of a particular cohort to assess cohort-level differences (pools information across sites), while the site -level random intercept allows for variation in productivity due to site conditions (pools information across cohorts). Priors for each cohort (*c*), and for each site (*s*) are drawn from global distributions to borrow strength across sites and cohorts.

$${\alpha }_{s} \sim normal({mu}_{\alpha }, {\sigma }_{\alpha s}^{2} )$$

$${\beta }_{1-6{\varvec{c}}} \sim normal({mu}_{\beta 1-6}, {\sigma }_{{\varvec{\beta}}1-6{\varvec{c}}}^{2})$$

$${mu}_{{\varvec{\beta}}1-6} \sim uniform(-2, 2)$$

$${mu}_{\alpha } \sim uniform(-2, 2)$$

$${\sigma }_{p}^{2} \sim inverse\, gamma(0.001, 0.001)$$

$${\sigma }_{\alpha s}^{2} \sim inverse\, gamma(0.001, 0.001)$$

$${\sigma }_{{\varvec{\beta}}1-6{\varvec{c}}}^{2} \sim inverse\, gamma(0.001, 0.001)$$

### *δ*^13^*C* and iWUE stable isotopes sampling design

We measured *δ*^13^*C* of α-cellulose and estimated yearly values for iWUE. *δ*^13^*C* of α-cellulose is recorded during photosynthesis, and thus is a good proxy for the internal CO_2_ concentrations present in the leaf stomata (C_i_) during carbon assimilation (McCarroll and Loader 2004). High atmospheric CO_2_ concentrations can elevate C_i_ by increasing ratio of CO_2_ uptake during assimilation (A) relative to water transpired (*g*_s_), driving increases in iWUE.

For each site, we identified ~ 20 years with similar climate conditions (10 years in each cohort that are most similar in terms of distance of PC1 and PC2 values of climate) to sample *δ*^13^*C* α-cellulose from 3 to 5 trees in each cohort (Table S4). We did this for 5 of our sites (BON, GLL1, GLL2, MOU, and UNC—see Table S4), resulting in 441 usable *δ*^13^*C* measurements used in the following analysis. This design allows us to compare *δ*^13^*C* and iWUE in years of similar climates, in similar sized trees, but under differing CO_2_ conditions.

We isolated α-cellulose from latewood following (Leavitt and Danzer 1993). ^13^C isotopic ratios were quantified by α-cellulose combustion in a Thermo DeltaV Advantage with a Costech 4010 EAS at University of Notre Dame Center for Environmental Science and Technology. Standard deviation for internal standards was ± 0.295 ‰. We use delta notation (*δ*^13^*C*) to indicate the ratio relative to the Vienna Pee Dee Belemnite global standard (‰ VPDB).

All *δ*^13^*C* estimates reported and modelled here are corrected for the Suess effect (Suess 1955), which accounts for recent changes in plant *δ*^13^*C* due to changes in atmospheric CO_2_ *δ*^13^*C* due to burning of fossil fuels (McCarroll and Loader 2004). iWUE is estimated using *δ*^13^*C* values and atmospheric CO_2_ concentrations (*c*_*a*_) from composite Mauna Loa observations and ice cores (Keeling et al. 2001). iWUE is determined using the following equation (as in Farquhar et al., 1982):

3 $$iWUE=\frac{A}{{g}_{s}}= \frac{{c}_{a}-{c}_{i}}{1.6}= \frac{{c}_{a} (b- {\Delta }^{13}C)}{1.6 (b-a)}$$

where *a* and *b* are the diffusion and Rubisco carboxylation fractionation factors (4.4 and 27 ‰), respectively. ${\Delta }^{13}C$ is isotopic discrimination, from measured δ^13^C atmospheric CO_2_ (*δ*^13^*C*_atm_) (Keeling et al. 2001) and *δ*^13^*C* of plant material (*δ*^13^*C*_plant_) (Farquhar et al. 1982):

4 $${\Delta }^{13}C= \frac{{\delta }^{13}{C}_{a}- {\delta }^{13}{C}_{plant}}{1+ \frac{{\delta }^{13}{C}_{plant}}{1000}}=a+(b-a)({c}_{i}/{c}_{a})$$

### Model selection and model description: iWUE and *δ*^13^*C*

We developed statistical models of WUE and *δ*^13^*C* that account for many of the non-CO_2_ factors can affect plant physiology. Specifically, interannual variations in high temperatures may decrease stomatal conductance (*g*_*s*_), and modify increases in iWUE (Granda et al. 2014; Guerrieri et al. 2019). Trends in *δ*^13^*C* and iWUE can also reflect moisture (Levesque et al. 2017)*,* tree size/age (Brienen et al. 2012; Vadeboncoeur et al. 2020), and stand structure (Granda et al., 2014). We model iWUE and *δ*^13^*C* as a function of climate, tree size, and stand structure. We include random intercepts ${\alpha }_{c}$ representative of “baseline” iWUE and *δ*^13^*C* for each* c* cohort, and cohort-level random slopes ${\beta }_{1-3c}$ on total precipitation (*Precip*), Maximum June temperature (*MaxTemp*), and tree size (*DBH*). As with the growth model, we fit a cohort-only and a cohort–structure model.

$${iWUE}_{ic} \sim normal\left(g\left(\alpha , \beta , Precip, DBH, MaxTemp\right)\right), {\sigma }_{p}^{2})$$

$${\delta^{13}C}_{ic} \sim normal\left(g\left(\alpha , \beta , Precip, DBH, MaxTemp\right)\right), {\sigma }_{p}^{2})$$

$$g={\alpha }_{c}+{\beta }_{1c}\times {Precip}_{i} +{\beta }_{2c}*{MaxTemp}_{i}+{\beta }_{3c}*{DBH}_{i}$$

$${\alpha }_{c} \sim normal({mu}_{\alpha }, {\sigma }_{\alpha c}^{2} )$$

$${\beta }_{1-3c} \sim normal({mu}_{\beta 1-3}, {\sigma }_{\beta 1-3c}^{2} )$$

$${mu}_{\beta 1-3} \sim uniform(-2, 2)$$

$${mu}_{\alpha } \sim unform(-2, 2)$$

$${\sigma }_{p}^{2} \sim inverse\, gamma(0.001, 0.001)$$

$${\sigma }_{\alpha c}^{2} \sim inverse\, gamma(0.001, 0.001)$$

$${\sigma }_{\beta 1-3c}^{2} \sim inverse\, gamma(0.001, 0.001)$$

After accounting for tree size, stand structure, and climate (${\beta }_{1-3c}$), the cohort differences between intercepts (${\alpha }_{c}$) represent the baseline differences in iWUE and *δ*^13^*C* likely resulting changing atmospheric CO_2_.

## Model implementation

Both the tree ring and stable isotope models were run for 275,000 MCMC iterations, thinning period of 15, and three chains, leaving posterior 18,334 MCMC samples. Convergence was determined based on visual inspection of mcmc plots and Gelman-Ruben diagnostic statistics (Gelman and Rubin 1992). All models were run in R using the rjags package (Plummer 2016).

### Future climate scenarios

We quantified a wide range of possible future climate conditions using a the mulitmodel ensemble of Climate Model Intercomparison Project 5 (CMIP5) projections (Table S7) (Reclamation 2013). We extracted the bias-corrected and statistically downscaled 1/8 degree resolution projections of maximum temperature and precipitation for our study sites, and summarized over the time periods 2025–2049, 2050–2075, and 2075–2099. Representative concentration pathways (RCP) 2.6, 4.5, 6.0, and 8.5 capture the range from low to high continued emissions and land-use scenarios. We use the range of future June maximum temperature and annual precipitation to characterize potential future conditions. To explore the consequences of future conditions, we evaluate posterior predictive responses o tree growth and the relative change in tree growth across historical temperature range (21–31 degrees C) under high (950 mm) and low (515 mm) annual precipitation values.

## Results

### Tree growth model

**Fig. 2:**
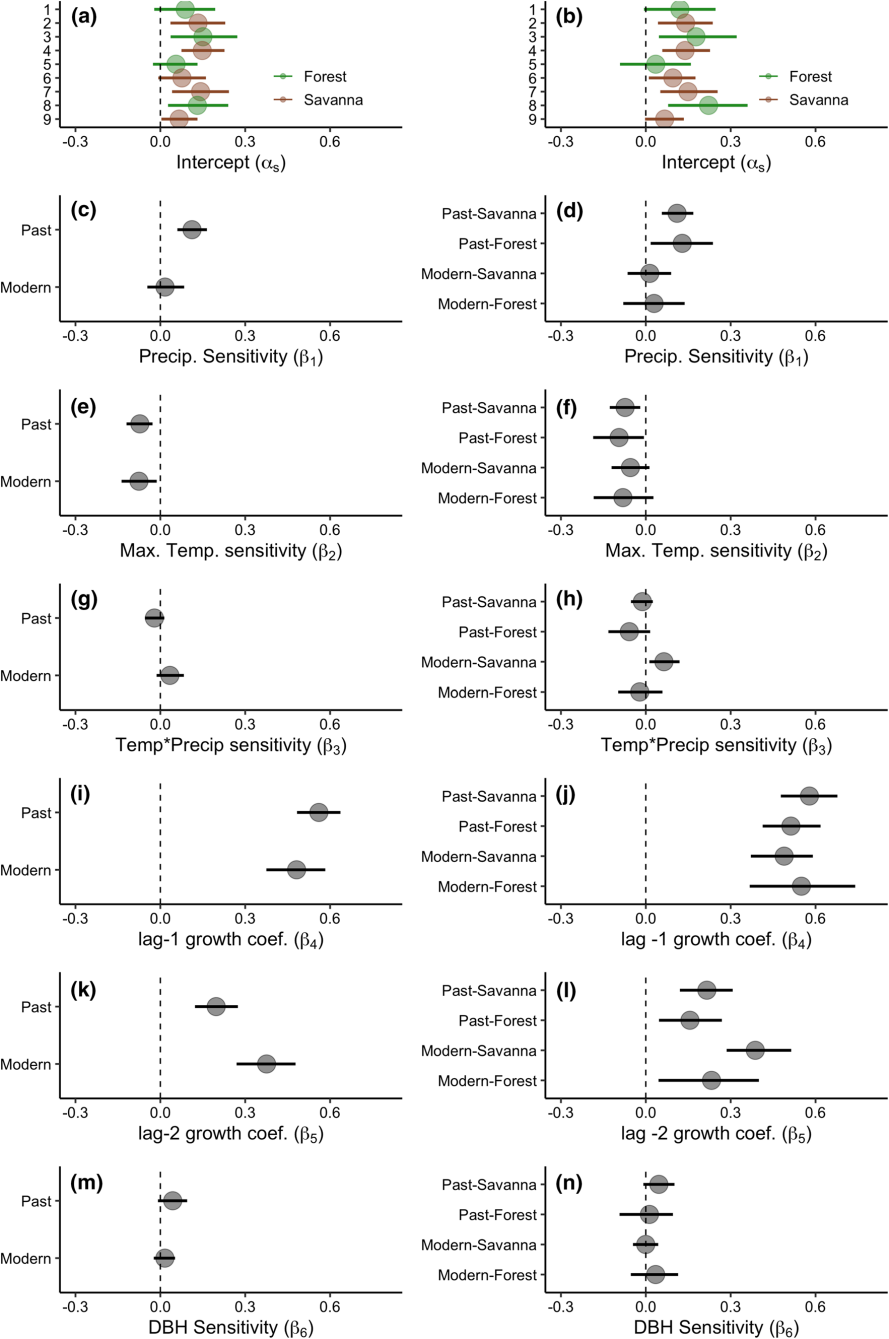
Coefficient estimates for tree ring growth models for cohort only (left panels; **a**, **c**, **e**, **g**, **i**, **k**, **m**) and cohort–structure models (right panels; **b**, **d**, **f**, **h**, **j**, **l**, **m**) for modern and past savanna and forest sites. **a**, **b** Plot-level random intercepts; **c**, **d** Precipitation sensitivity; **e**, **f** Maximum temperature sensitivity; **g**, **h** Temperature*Precipitation sensitivity; **i**, **j** Effect of previous year’s growth (lag-1 sensitivity); **k**, **l** Effect of the growth 2 years prior (lag 2 sensitivity); **m**, **n** Effect of DBH. Circles indicate mean estimates, bars are 95% credible intervals

The cohort-only random effects and the structure–cohort random effects models both track observed growth (cohort-only model *R*^2^ = 0.729; structure–cohort model *R*^2^ = 0.739) (Fig. S6 A-B), and capture interannual and site level differences observed in the raw data (Fig. S7). Although adding structure effects to the model only marginally improves model fit, we discuss the results of both models below, as the structure–cohort model provides a more detailed assessment of factors affecting tree growth. Site-level random intercepts show differences in mean values of predicted tree-ring growth (Fig. 2a, b, Table S3), but are not well explained by factors such as temperature, precipitation, average tree size, number of species, or saturated hydraulic conductivity, or soil water content.

### Are there changes in precipitation sensitivity over the twentieth century?

Past tree growth in both savannas and forests is positively related to total annual precipitation, with a *β*_precip_ sensitivities of 0.111 (95% CI 0.06–0.17) and 0.129 (95% CI 0.02–0.24), respectively (Fig. 2c, d, Table S3). In contrast, modern trees are not significantly sensitive to precipitation in either savannas (*β*_precip_ = 0.014, 95% CI − 0.06–0.09) or closed forests (*β*_precip_ = 0.029, 95% CI − 0.08–0.14) (Fig. 2c, d, Table S3), representing a 69% (95% CI 24–116%) decrease in *β*_precip_ sensitivity (Fig. S8C). This decline is stronger in savannas than in closed forests (Fig. 2d, Fig. S13). Conditioned on means for all other covariates, the decreased precipitation sensitivity in the modern cohort results in a growth benefit under low precipitation conditions of about 20% (95% CI 3–34%) in savannas and 33% (95% CI − 3–99%) in closed forests (Fig. S13).

### Do higher temperatures have a negative effect on tree growth?

**Fig. 3:**
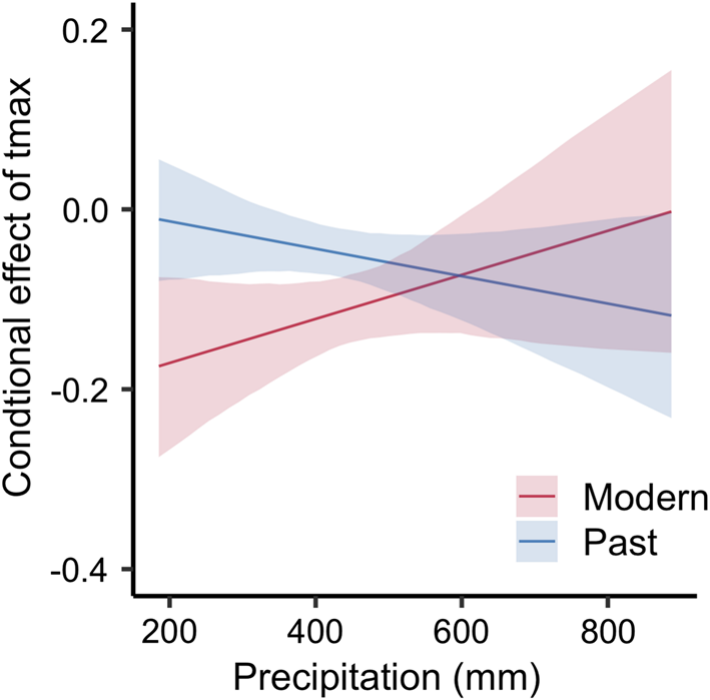
Effect of June maximum temperature (*T*max) on tree growth conditioned on precipitation, and mean values for all covariates. The interaction is significant for the modern cohort, but not for the past cohort

June maximum temperature negatively affects tree growth in the cohort only model, with a past *β*_tmax_ of -0.072 (95% CI − 0.11–0.03) and modern *β*_tmax_ of -0.076 (95% CI − 0.14–0.01) (Fig. 2e, f, Table S3). But, the modern cohort has an interaction between maximum temperature and precipitation (Fig. 2g, h, Table S3), such that high temperatures negatively impact growth at low precipitations, but positively affect growth when precipitation surpasses ~ 800 mm/year, which only occurs in < 10% of the years in our dataset (Fig. 3).

The negative effect of June maximum temperature on tree growth persists across all stand structures and cohorts. Past savannas and forests have a *β*_tmax_ of − 0.073 (95% CI − 0.13 to − 0.02) and − 0.094 (95% CI − 0.19 to − 0.01), respectively (Fig. 2F, Table S3). Modern savannas and forests have similar sensitivities, with *β*_tmax_ of -0.054 (95% CI − 0.12 to 0.01) and -0.081 (95% CI − 0.18–0.03), respectively. The precipitation and temperature interaction is strongest in modern savanna stand structures (*β*_tmax_ = 0.064 (95% CI 0.01–0.12)) (Fig. 2g, h, Table S3, Fig. S14).

### Effects of previous years’ growth on tree growth

For both models, lag-1 parameters exert a strong positive effect on tree growth across stand structures and age classes (Past Savanna *β*_lag-1_ = 0.578 (95% CI 0.48–0.68), Modern Savanna *β*_lag-1_ = 0.489 (95% CI 0.37–0.59), Past Forest *β*_lag-1_ = 0.513 (95% CI 0.41–0.62), Modern Forest *β*_lag-1_ = 0.55 (95% CI 0.37–0.74)) (Fig. 2i, j, Table S3).

For both the cohort only and the structure–cohort models, the lag-2 autocorrelation parameters vary such that modern trees are more strongly correlated to growth two years prior compared to their past counterparts, and savanna systems have higher lag-2 parameter estimates overall (Past Savanna *β*_lag-2_ = 0.216 (95% CI 0.12–0.31), Modern Savanna *β*_lag-2_ = 0.387 (95% CI 0.29–0.51), Past Forest *β*_lag-2_ = 0.129 (95% CI 0.05–0.27), Modern Forest *β*_lag-2_ = 0.232 (95% CI 0.04–0.4)) (Fig. 2k, l, Table S3). Finally, tree diameter generally has a positive effect on tree growth in both the cohort-only and cohort–structur*e* models (Fig. 2m, n, Table S3).

### Does increased CO_2_ over the twentieth century result in increases in tree growth?

On average, we do not detect significant differences in the posterior predicted tree growth across cohorts, with average tree growth for the past cohort at 1.37 mm/year (95% CI 0.36–3.56 mm/year) and 1.68 (95% CI 0.319–4.93 mm/year) for the modern cohort (Fig. S8B). There are no significant differences in average growth between modern and past cohorts across stand structure (Fig. S12), though modern forests may be trending towards increased growth with ~ 1 mm higher average growth than past forests.

### WUE and *δ*^13^*C* models

The cohort-only random effects and the structure–cohort models of iWUE both captured the observed values of iWUE (cohort-only model *R*^2^ = 0.349; structure–cohort model *R*^2^ = 0.386) (Fig. S10). The *δ*^13^*C* cohort-only model has high prediction error in reconstructing observed *δ*^13^*C* (*R*^2^ = 0.086), but the structure–cohort model improves prediction (*R*^2^ = 0.196) (Fig. S11). Due to poor *δ*^13^*C* cohort-only model fit (Fig. S11), we focus on the results of iWUE and *δ*^13^*C* cohort–structure models in the main text, but report the cohort-only models in Table S5.

### Does WUE increase with increased atmospheric CO_2_?

**Fig. 4:**
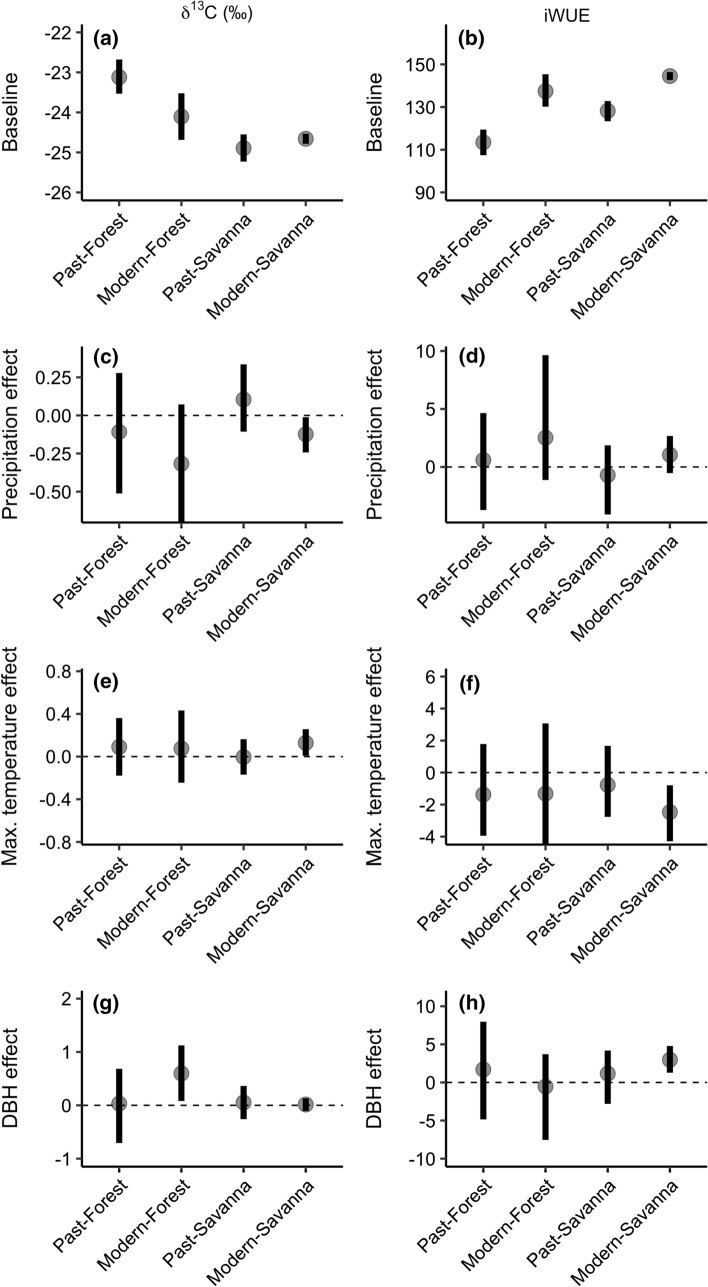
Cohort–structure model estimates for δ^13^C (left column) and iWUE (right column) models. **a**, **b** Baseline α coefficients, **c**, **d** precipitation effect, **e**, **f** June maximum temperature effect, **g**, **h** tree diameter (DBH) effect

Predicted iWUE increases by about 23.1% in closed forests between the modern and past cohorts, but only increases by 16.4% in open savanna sites (Fig. S8A). Baseline iWUE increases are also smaller in savannas, with a 12.8% increase (95% CI 8.6–17.5%), compared to closed forests, which increased by 21% (95% CI 12.3–30.9%) (Fig. 4b). Baseline *δ*^13^*C* values support this pattern; closed forests baseline *δ*^13^*C* became more negative by 0.99‰ (95% CI − 1.72 to − 2.8‰)*,* while open savannas became less negative by 0.23‰ (95% CI − 0.13–0.59‰) in baseline *δ*^13^*C* (Fig. 4a, Table S5). However, past savanna *δ*^*13*^*C* is much more negative than that of past closed forests (*δ*^13^*C*_savanna_ = − 24.894 ‰ (95% CI − 25.23 to − 24.55‰), *δ*^13^*C*_forest_ = − 23.119‰ (95% CI − 22.53 to − 23.53‰)), and iWUE is higher in past savannas compared to closed forests, indicating that stand structure is a strong modifier of physiological responses (Fig. 4a, b).

### Physiological effects of climate and tree size on estimated *δ*^13^*C* and iWUE

Precipitation has slight negative effect on *δ*^13^*C*, but the 95% CI of *β*_precip_ overlaps with zero in most structure and cohort groups (Fig. 4c, Table S5). Precipitation effect on iWUE is overlapping with 0 for estimates across all stand structure and cohort types (Past Savanna *β*_precip_ = − 0.706 (95% CI − 4.1–1.86), Modern Savanna *β*_precip_ = 1.044 (95% CI − 0.53–2.68), Past Forest *β*_precip_ = 0.592 (95% CI − 3.72–4.65), Modern Forest *β*_precip_ = 2.52 (95% CI − 1.13–9.64)) (Fig. 4d, Table S5). A significant positive response of δ^13^C to June maximum temperature occurs in the modern open savanna cohort (*β*_tmax_ = 0.128 (95% CI 0.01–0.26)), possibly due to stomatal closure under high temperatures (Fig. 4e, Table S5). This results in a negative temperature effect on iWUE in modern savanna systems (*β*_tmax_ = − 2.47 (95% CI − 4.29 to − 0.79)(Fig. 4f). Finally, tree diameter had little effect on estimates of iWUE, with the exception of Modern savanna systems (2.976 95% CI 1.28–4.78), likely because we focused on trees of similar size (20–40 cm) in isotope analyses (Fig. 4g, h, Table S5).

### Growth responses under future climate

**Fig. 5:**
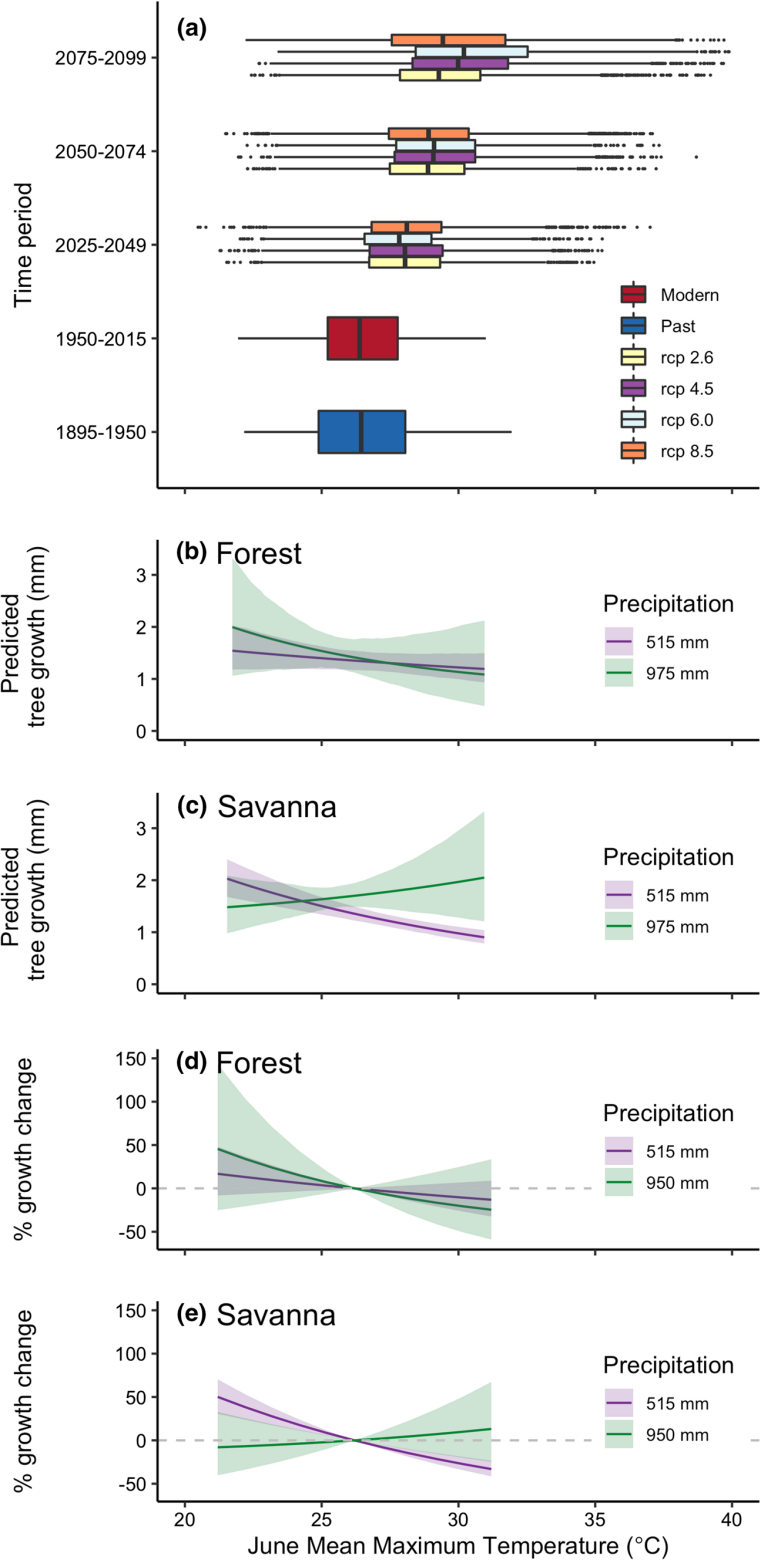
Predicted effects of climate changes on tree growth of forest and savanna trees. **a** Past, modern, and future June maximum temperatures projected by CMIP5 models under rcps 2.6, 4.5, 6.0, and 8.5. **b**, **c** Posterior predicted tree-growth responses to June tmax under low and high precipitation (515 mm and 950 mm) for **b** forests and **c** savannas, holding all other covariates at mean values. **d**, **e**. Percent difference in tree growth relative to the regional median temperature (26 °C) condition, for **d** forests and **c** savannas

Projections from CMIP5 show modest warming of ~ 1.5–28 °C in the near future (2025–2049), with high interannual, intermodal, and rcp scenario variation (range: 20.5–37 °C) (Fig. 5a). Posterior predictive distributions of tree growth based on in-sample historical climate space indicate that effects of high future temperatures will depend strongly on stand structure and precipitation (Fig. 5b–e). When precipitation is higher than the historical averages (975 mm), high temperatures result in ~ 20% growth decline in forests, but with high uncertainty (95% CI − 54–42%) (Fig. 5d). Savannas also have high uncertainty under these conditions, but show smaller changes, (95% CI – 25–46%). However, in dry conditions (annual precipitation = 515 mm) and high temperatures, tree growth declines are predicted for both forests and savannas. Savannas have a much stronger negative response to temperature, with  − 34% (95% CI − 47 to − 23%) decreases in tree growth (Fig. 5e).

Projected temperatures increase by ~ 2.5 °C by 2050–2074, and by 2075–2099, projected temperatures increase by ~ 3 °C, to 29.4–30.5 °C (Range 22.2–40.9 °C). With many projected temperatures falling outside of the historical range (Fig. 5a), it is difficult to quantify the impacts on tree growth without extrapolating. However, general trends of tree growth declines indicate that higher temperatures will have a large negative effects on growth (Fig. 5).

## Discussion

We document increases in iWUE and decreases in drought sensitivity that are consistent with a positive effect of CO_2_ on tree function in the Upper Midwest, but these changes do not result in detectable increases in tree growth. The potential benefits of iWUE do not translate to tree growth, as tree growth is limited by an interaction between precipitation and temperature in the modern cohort, which causes declines of up to ~ 50% growth under hot and dry conditions (Fig. 5). This effect is strongest in savannas, indicating that stand structure can amplify responses to climate change.

### WUE increases, but does not increase tree growth

iWUE increased by 16–23% over the past century, but does not result in a detectable net increase in radial tree growth. This is consistent with tree ring iWUE evidence from across the globe, which estimate increases of ~ 15–30% over the twentieth century, but rarely report concurrent increases tree growth (Peñuelas et al. 2011; Andreu‐Hayles et al. 2011; Tognetti et al. 2014; van der Sleen et al. 2015; Frank et al. 2015; Fernández‐de‐Uña et al. 2016; Giguère-Croteau et al. 2019; Guerrieri et al. 2019). In jointly modelling the effects of CO_2_ and climate on tree-ring growth and physiological responses, we gain insight into possible mechanisms for why increases in iWUE do not lead to changes in tree-ring growth (Fig. S8B). Below, we discuss possible reasons for this paradox supported by our analysis: decreased precipitation sensitivity, higher temperature stress, and correlation with previous years’ growth.

### Precipitation sensitivity shifts consistent with benefits of higher iWUE

Reduced precipitation sensitivity observed here is consistent with previous evidence from the region (Wyckoff and Bowers 2010; Maxwell et al. 2016). This reduction is hypothesized to be due to higher CO_2_ and iWUE, or a result of increased iWUE due to more moist climatic conditions (Maxwell et al. 2016; Levesque et al. 2017). Declining precipitation sensitivity is consistent both overall (Fig. S5), and during the driest years (Fig. 2), which were modeled to reduce the potential effect of recent pluvial conditions. Though, effects of long-term moisture trends are difficult to quantify and may still play a role in shifting drought sensitivity. Regardless of the cause, lower precipitation sensitivity yields non-trivial growth benefits in dry conditions. Conditioned on other covariates, lower precipitation sensitivity provides savannas a ~ 45% increase in growth during dry years and a ~ 30% increase in closed forests (Fig. S13). However, these effects are strongly context dependent, which is likely why there are no detectable increases in annual tree growth overall (Figs. 5 and S5).

We also document the rising importance of previous years’ growth on annual growth increment, which we hypothesize could also be a product of feedbacks between growth and changes in carbon allocation with elevated iWUE. High CO_2_ conditions promote allocation to non-radial growth tissues (Zweifel and Sterck 2018; Kannenberg et al. 2019) that are not observable using tree-ring data alone. For example, increased CO_2_ can drive enhanced root or leaf production to improve resource acquisition (Norby and Zak 2011) and drive non-structural carbohydrate production (Wullschleger et al. 1992), both of which could have lagged effects on tree growth (Zweifel and Sterck 2018; Kannenberg et al. 2019). Lagged effects are also observed elsewhere in confers, and could result from changes in leaf/needle turnover and growth, antecedent climate, and limited opportunity to use stored carbon (Littell et al. 2008). Antecedent climate conditions, such as recent pluvial conditions (Helcoski et al. 2019), and drought legacies (Ogle et al. 2015; Szejner et al. 2018), could be driving increased lagged effects, as well as the reduced precipitation sensitivities. Regardless of underlying mechanism, large lagged effects would compound growth declines under multi-year drought events or amplify growth increases under multi-year pluvial events.

### Summer temperature has an increasing impact on growth, iWUE, and *δ*^13^*C*

Our hierarchical model shows that while elevated CO_2_ and iWUE relieves moisture limitations under specific contexts (Fig. S13), these positive effects are overwhelmed by increased temperature stress. Under hot and wet conditions, modern trees have either equal or slightly increased growth compared to similar trees in the past, but suffer substantially greater growth declines under hot and dry conditions (Fig. 3). Limitation by higher summer temperatures could help explain why increased CO_2_ and iWUE do not result in the expected overall increases in tree growth (Farquhar et al. 1982; Ainsworth and Long 2005). Indeed, elevated CO_2_ experiments show that limiting factors can diminish the effects of enhanced CO_2_ (Ainsworth and Long 2005; Finzi et al. 2006). Recent evidence also suggests that reduced stomatal conductance in dry conditions may drive recent iWUE changes in the Eastern US (Guerrieri et al. 2019), and high temperatures are increasingly cited as drivers of tree growth declines and mortality (Breshears et al. 2005; Adams et al. 2009; Allen et al. 2015).

Shifts in the temperature sensitivities of *δ*^13^*C* and iWUE, particularly in modern savannas, provide additional physiological evidence for increased temperature limitations on carbon assimilation. Low moisture and high temperature stress can either drive water loss, xylem cavitation, or drive stomatal closure and reduced photosynthesis (Zhou et al. 2013). Extended stomatal closures can limit both carbon assimilation and conductance, leading to increased *δ*^13^*C* signatures as discrimination decreases (Farquhar et al. 1982). The heightened *δ*^13^*C* and iWUE sensitivity to temperatures in modern savannas (Fig. 4e, f) could indicate stomatal closure under temperature-induced drought stress, identifying a candidate physiological mechanism driving the temperature and precipitation interaction in the growth model. This interaction is consistent with the well documented role that rising temperatures and increased Vapor Pressure Deficit (VPD) plays driving reductions in tree growth and forest productivity (Adams et al. 2009; Williams et al. 2020). If modern cohort responses are any indication of future responses in the region, then elevated temperature and VPD stress, not elevated precipitation or CO_2_, will be the dominant constraint on tree growth and physiology in the future.

### Stand structure modulates climate and physiological responses

Stand structure strongly shapes the recent changes in climate sensitivity and iWUE. Savannas had higher growth, *δ*^13^*C*, and iWUE sensitivities to temperature, consistent with both previous work showing physiological responses to different light and microclimate conditions (Litvak et al. 1996; Monson et al. 2016; Frey et al. 2016; Reinmann and Hutyra 2017) and our hypothesis that open canopy microclimates create hotter and drier conditions that amplify negative effects temperature. However, this contrasts studies showing that competition at high tree densities leads to growth declines and mortality (Gea-Izquierdo et al. 2009; Fernández-de-Uña et al. 2015). Our analysis highlights that differences between savanna and forest climate sensitivity have become more pronounced over time, and that future temperature increases will have adverse physiological and growth effects in open canopy microclimates.

### Future growth responses

Whether the hypothesized benefits of high CO_2_ and iWUE can offset negative impacts of climate change is a key uncertainty in future distribution and carbon balance in terrestrial systems (Friedlingstein et al. 2013). Here, we show that the benefits of higher iWUE could improve *Quercus spp.* growth under cool and dry climate conditions (Fig. S13, Fig. 5). However, we also see substantial growth declines under hot and dry conditions, indicating that futures temperature will drive future regional responses (Fig. 5). Furthermore, increased reliance on previous years’ growth observed here could amplify negative impacts of high temperatures.

If the extensive tree-growth declines projected here lead to tree mortality, then more frequent and hotter future droughts (Clark et al. 2016) could lead to shifts in distribution of open and closed systems both here (Iverson et al. 2008; Adams et al. 2009; Frelich and Reich 2010), and worldwide (Anadón et al. 2014; Moncrieff et al. 2016)*. *While reduced growth does not necessarily result in mortality, the conditions driving growth declines here (high temperature, low precipitation) are the same stressors that drive both xylem damage in *Quercus spp*. and tree mortality (Breshears et al. 2005; Adams et al. 2009; Allen et al. 2015). Hotter droughts may trigger tree mortality faster than cool droughts and could drive rapid tree die-off (Adams et al. 2009; Allen et al. 2015). Mortality could further open the canopy, creating a “death spiral” that increases tree stress and mortality (Franklin et al. 1987; Allen and Breshears 1998; Breshears et al. 2005). Thus, there is urgent need to quantitatively forecast changes in tree-growth and physiology, such as those documented here, and how they scale up to population changes in tree mortality, community dynamics, and ecosystem-scale feedbacks.

## Conclusions

While the changes in iWUE and CO_2_ that occurred during the twentieth century did not result in a detectable net increase in radial tree growth in the Midwestern US, we document both altered growth responses to climate and increased autocorrelation in tree-ring growth. Although there is reduced precipitation stress across the region, these same trees now experience greater stress, resulting in large tree growth declines as temperatures rise. These effects are strongest in open savannas, indicating that canopy microclimatic feedbacks are important modifiers of climate change responses. Interactive effects between temperature and precipitation in different canopy microclimates are often not explored extensively when forecasting tree-ring growth responses, but could make systems vulnerable to future vegetation shifts.

## Supplementary Information

Below is the link to the electronic supplementary material.

Supplementary file1 (PDF 6279 KB)

## Data Availability

Data used for analysis in this manuscript is archived at EDI data portal (https://portal.edirepository.org/nis/), package ids: msb-paleon.34.0, msb-paleon.35.0, msb-paleon.36.0, msb-paleon.37.0, msb-paleon.38.0, msb-paleon.39.0, msb-paleon.40.0, msb-paleon.41.0, msb-paleon.42.0, msb-paleon.43.0. Code is available on GitHub (https://github.com/Kah5/TreeRings).

## References

[CR1] Adams HD, Guardiola-Claramonte M, Barron-Gafford GA, Villegas JC, Breshears DD, Zou CB, Troch PA, Huxman TE, Mooney HA (2009). Temperature sensitivity of drought-induced tree mortality portends increased regional die-off under global-change-type drought. Proc Natl Acad Sci USA.

[CR2] Ainsworth EA, Long SP (2005). What have we learned from 15 years of free-air CO2 enrichment (FACE)? A meta-analytic review of the responses of photosynthesis, canopy properties and plant production to rising CO2. New Phytol.

[CR3] Allen CD, Breshears DD (1998). Drought-induced shift of a forest-woodland ecotone: rapid landscape response to climate variation. Proc Natl Acad Sci USA.

[CR4] Allen CD, Breshears DD, McDowell NG (2015). On underestimation of global vulnerability to tree mortality and forest die-off from hotter drought in the Anthropocene. Ecosphere.

[CR5] Anadón JD, Sala OE, Maestre FT (2014). Climate change will increase savannas at the expense of forests and treeless vegetation in tropical and subtropical Americas. J Ecol.

[CR6] Andreu-Hayles L, Planells O, Gutiérrez E, Muntan E, Helle G, Anchukaitis KJ, Schleser GH (2011). Long tree-ring chronologies reveal 20th century increases in water-use efficiency but no enhancement of tree growth at five Iberian pine forests. Glob Change Biol.

[CR7] Babst F, Alexander MR, Szejner P, Bouriaud O, Klesse S, Roden J, Ciais P, Poulter B, Frank D, Moore DJP, Trouet V (2014). A tree-ring perspective on the terrestrial carbon cycle. Oecologia.

[CR8] Bowman DMJS, Brienen RJW, Gloor E, Phillips OL, Prior LD (2013). Detecting trends in tree growth: not so simple. Trends Plant Sci.

[CR9] Breshears DD, Cobb NS, Rich PM, Price KP, Allen CD, Balice RG, Romme WH, Kastens JH, Floyd ML, Belnap J, Anderson JJ, Myers OB, Meyer CW (2005). Regional vegetation die-off in response to global-change-type drought. Proc Natl Acad Sci USA.

[CR10] Brewer P (2014). Data management in dendroarchaeology using tellervo. Radiocarbon.

[CR11] Brienen RJW, Gloor E, Zuidema PA (2012). Detecting evidence for CO2 fertilization from tree ring studies: The potential role of sampling biases. Global Biogeochem Cycles.

[CR12] Brienen RJW, Gloor E, Clerici S, Newton R, Arppe L, Boom A, Bottrell S, Callaghan M, Heaton T, Helama S, Helle G, Leng MJ, Mielikäinen K, Oinonen M, Timonen M (2017). Tree height strongly affects estimates of water-use efficiency responses to climate and CO 2 using isotopes. Nat Commun.

[CR13] Brienen RJW, Gloor M, Ziv G (2017). Tree demography dominates long-term growth trends inferred from tree rings. Glob Change Biol.

[CR14] Bunn AG (2010). Statistical and visual crossdating in R using te dplR library. Dendrochronologia.

[CR15] Charney ND, Babst F, Poulter B, Record S, Trouet VM, Frank D, Enquist BJ, Evans MEK (2016). Observed forest sensitivity to climate implies large changes in 21st century North American forest growth. Ecol Lett.

[CR16] Chen J, Franklin JF, Spies TA (1993). Contrasting microclimates among clearcut, edge, and interior of old-growth Douglas-fir forest. Agric For Meteorol.

[CR17] Clark JS, Iverson L, Woodall CW, Allen CD, Bell DM, Bragg DC, D’Amato AW, Davis FW, Hersh MH, Ibanez I, Jackson ST, Matthews S, Pederson N, Peters M, Schwartz MW, Waring KM, Zimmermann NE (2016). The impacts of increasing drought on forest dynamics, structure, and biodiversity in the United States. Glob Change Biol.

[CR18] Cook E (1990). Methods of Dendrochronology—Applications in the Environmental Sciences.

[CR19] Cook BI, Ault TR, Smerdon JE (2015). Unprecedented 21st century drought risk in the American Southwest and Central Plains. Sci Adv.

[CR20] Davis SC, Hessl AE, Scott CJ, Adams MB, Thomas RB (2009). Forest carbon sequestration changes in response to timber harvest. For Ecol Manage.

[CR21] Dietze M (2017). Ecological Forecasting.

[CR22] Druckenbrod DL, Martin-Benito D, Orwig DA, Pederson N, Poulter B, Renwick KM, Shugart HH (2019). Redefining temperate forest responses to climate and disturbance in the eastern United States: new insights at the mesoscale. Glob Ecol Biogeogr.

[CR23] Dye A, Plotkin AB, Bishop D, Pederson N, Poulter B, Hessl A (2016). Comparing tree-ring and permanent plot estimates of aboveground net primary production in three eastern U.S. forests. Ecosphere.

[CR24] Farquhar GD, O’Leary MH, Berry JA (1982). On the relationship between carbon isotope discrimination and the intercellular carbon dioxide concentration in leaves. Functional Plant Biol.

[CR25] Fernández-de-Uña L, Cañellas I, Gea-Izquierdo G (2015). Stand competition determines how different tree species will cope with a warming climate. PLoS ONE.

[CR26] Fernández-de-Uña L, McDowell NG, Cañellas I, Gea-Izquierdo G (2016). Disentangling the effect of competition, CO2 and climate on intrinsic water-use efficiency and tree growth. J Ecol.

[CR27] Finzi AC, Moore DJP, DeLucia EH, Lichter J, Hofmockel KS, Jackson RB, Kim H-S, Matamala R, McCarthy HR, Oren R, Pippen JS, Schlesinger WH (2006). Progressive nitrogen limitation of ecosystem processes under elevated Co2 in a warm-temperate forest. Ecology.

[CR28] Foster JR, Finley AO, D’Amato AW, Bradford JB, Banerjee S (2016). Predicting tree biomass growth in the temperate-boreal ecotone: Is tree size, age, competition, or climate response most important?. Glob Chang Biol.

[CR29] Frank DC, Poulter B, Saurer M, Esper J, Huntingford C, Helle G, Treydte K, Zimmermann NE, Schleser GH, Ahlström A, Ciais P, Friedlingstein P, Levis S, Lomas M, Sitch S, Viovy N, Andreu-Hayles L, Bednarz Z, Berninger F, Boettger T, D’Alessandro CM, Daux V, Filot M, Grabner M, Gutierrez E, Haupt M, Hilasvuori E, Jungner H, Kalela-Brundin M, Krapiec M, Leuenberger M, Loader NJ, Marah H, Masson-Delmotte V, Pazdur A, Pawelczyk S, Pierre M, Planells O, Pukiene R, Reynolds-Henne CE, Rinne KT, Saracino A, Sonninen E, Stievenard M, Switsur VR, Szczepanek M, Szychowska-Krapiec E, Todaro L, Waterhouse JS, Weigl M (2015). Water-use efficiency and transpiration across European forests during the Anthropocene. Nature Clim Change.

[CR30] Franklin JF, Shugart HH, Harmon ME (1987). Tree death as an ecological process. BioScience.

[CR31] Freckleton RP (2002). On the misuse of residuals in ecology: regression of residuals vs. multiple regression. J Anim Ecol.

[CR32] Frelich LE, Reich PB (2010). Will environmental changes reinforce the impact of global warming on the prairie–forest border of central North America?. Front Ecol Environ.

[CR33] Frey SJK, Hadley AS, Johnson SL, Schulze M, Jones JA, Betts MG (2016). Spatial models reveal the microclimatic buffering capacity of old-growth forests. Sci Adv.

[CR34] Friedlingstein P, Meinshausen M, Arora VK, Jones CD, Anav A, Liddicoat SK, Knutti R (2013). Uncertainties in CMIP5 climate projections due to carbon cycle feedbacks. J Climate.

[CR35] Gea-Izquierdo G, Martín-Benito D, Cherubini P, Cañellas I (2009). Climate-growth variability in *Quercus ilex* L. west Iberian open woodlands of different stand density. Ann For Sci.

[CR36] Gelman A, Rubin DB (1992). Inference from iterative simulation using multiple sequences. Statist Sci.

[CR37] Giguère-Croteau C, Boucher É, Bergeron Y, Girardin MP, Drobyshev I, Silva LCR, Hélie J-F, Garneau M (2019). North America’s oldest boreal trees are more efficient water users due to increased [CO2], but do not grow faster. Proc Natl Acad Sci USA.

[CR38] Goring SJ, Mladenoff DJ, Cogbill CV, Record S, Paciorek CJ, Jackson ST, Dietze MC, Dawson A, Matthes JH, McLachlan JS, Williams JW (2016). Novel and lost forests in the upper Midwestern United States, from new estimates of settlement-era composition, stem density, and biomass. PLoS ONE.

[CR39] Granda E, Rossatto DR, Camarero JJ, Voltas J, Valladares F (2014). Growth and carbon isotopes of Mediterranean trees reveal contrasting responses to increased carbon dioxide and drought. Oecologia.

[CR40] Guerrieri R, Belmecheri S, Ollinger SV, Asbjornsen H, Jennings K, Xiao J, Stocker BD, Martin M, Hollinger DY, Bracho-Garrillo R, Clark K, Dore S, Kolb T, Munger JW, Novick K, Richardson AD (2019). Disentangling the role of photosynthesis and stomatal conductance on rising forest water-use efficiency. PNAS.

[CR41] Hararuk O, Campbell EM, Antos JA, Parish R (2019). Tree rings provide no evidence of a CO_2_ fertilization effect in old-growth subalpine forests of western Canada. Glob Change Biol.

[CR42] Helcoski R, Tepley AJ, Pederson N, McGarvey JC, Meakem V, Herrmann V, Thompson JR, Anderson-Teixeira KJ (2019). Growing season moisture drives interannual variation in woody productivity of a temperate deciduous forest. New Phytol.

[CR43] Hobbs NT, Hooten MB (2015). Bayesian models: a statistical primer for ecologists.

[CR44] Iverson LR, Prasad AM, Matthews SN, Peters M (2008). Estimating potential habitat for 134 eastern US tree species under six climate scenarios. For Ecol Manage.

[CR45] Kannenberg SA, Novick KA, Alexander MR, Maxwell JT, Moore DJP, Phillips RP, Anderegg WRL (2019). Linking drought legacy effects across scales: from leaves to tree rings to ecosystems. Glob Change Biol.

[CR46] Keeling CD, Piper SC, Bacastow RB, Wahlen M, Whorf TP, Heimann M, Meijer HA (2001) Exchanges of Atmospheric CO2 and 13CO2 with the Terrestrial Biosphere and Oceans from 1978 to 2000. I. Global Aspects. Scripps Institution of Oceanography

[CR47] Keenan TF, Hollinger DY, Bohrer G, Dragoni D, Munger JW, Schmid HP, Richardson AD (2013). Increase in forest water-use efficiency as atmospheric carbon dioxide concentrations rise. Nature.

[CR48] Klesse S, DeRose RJ, Guiterman CH, Lynch AM, O’Connor CD, Shaw JD, Evans MEK (2018). Sampling bias overestimates climate change impacts on forest growth in the southwestern United States. Nat Commun.

[CR49] Leavitt SW, Danzer SR (1993). Method for batch processing small wood samples to holocellulose for stable-carbon isotope analysis. Anal Chem.

[CR50] Levesque M, Andreu-Hayles L, Pederson N (2017). Water availability drives gas exchange and growth of trees in northeastern US, not elevated CO_2_ and reduced acid deposition. Sci Rep.

[CR51] Littell JS, Peterson DL, Tjoelker M (2008). Douglas-fir growth in mountain ecosystems: water limits tree growth from stand to region. Ecol Monogr.

[CR52] Litvak ME, Loreto F, Harley PC, Sharkey TD, Monson RK (1996). The response of isoprene emission rate and photosynthetic rate to photon flux and nitrogen supply in aspen and white oak trees. Plant, Cell Environ.

[CR53] Maxwell JT, Harley GL, Robeson SM (2016). On the declining relationship between tree growth and climate in the Midwest United States: the fading drought signal. Clim Change.

[CR54] McCarroll D, Loader NJ (2004). Stable isotopes in tree rings. Quatern Sci Rev.

[CR55] Moncrieff GR, Scheiter S, Langan L, Trabucco A, Higgins SI (2016). The future distribution of the savannah biome: model-based and biogeographic contingency. Phil Trans R Soc B.

[CR56] Monson RK (2003). The many faces of plant carbon relations: forging an ecophysiological identity in the age of human influence. New Phytol.

[CR57] Monson RK, Neice AA, Trahan NA, Shiach I, McCorkel JT, Moore DJP (2016). Interactions between temperature and intercellular CO2 concentration in controlling leaf isoprene emission rates. Plant Cell Environ.

[CR58] Nolan C, Overpeck JT, Allen JRM, Anderson PM, Betancourt JL, Binney HA, Brewer S, Bush MB, Chase BM, Cheddadi R, Djamali M, Dodson J, Edwards ME, Gosling WD, Haberle S, Hotchkiss SC, Huntley B, Ivory SJ, Kershaw AP, Kim S-H, Latorre C, Leydet M, Lézine A-M, Liu K-B, Liu Y, Lozhkin AV, McGlone MS, Marchant RA, Momohara A, Moreno PI, Müller S, Otto-Bliesner BL, Shen C, Stevenson J, Takahara H, Tarasov PE, Tipton J, Vincens A, Weng C, Xu Q, Zheng Z, Jackson ST (2018). Past and future global transformation of terrestrial ecosystems under climate change. Science.

[CR59] Norby RJ, Zak DR (2011). Ecological lessons from free-air CO2 enrichment (FACE) experiments. Annu Rev Ecol Evol Syst.

[CR60] Ogle K, Barber JJ, Barron-Gafford GA, Bentley LP, Young JM, Huxman TE, Loik ME, Tissue DT (2015). Quantifying ecological memory in plant and ecosystem processes. Ecol Lett.

[CR61] Olson DM, Dinerstein E, Wikramanayake ED, Burgess ND, Powell GVN, Underwood EC, D’amico JA, Itoua I, Strand HE, Morrison JC, Loucks CJ, Allnutt TF, Ricketts TH, Kura Y, Lamoreux JF, Wettengel WW, Hedao P, Kassem KR (2001). Terrestrial ecoregions of the world: a new map of life on EarthA new global map of terrestrial ecoregions provides an innovative tool for conserving biodiversity. Bioscience.

[CR62] Peñuelas J, Canadell JG, Ogaya R (2011). Increased water-use efficiency during the 20th century did not translate into enhanced tree growth. Glob Ecol Biogeogr.

[CR63] Plummer M (2016) rjags: Bayesian Graphical Models using MCMC

[CR64] PRISM Climate Group, Oregon State University (2004) http://prism.oregonstate.edu. Accesed 22 Oct 2019

[CR65] Reclamation B of (2013). Downscaled CMIP3 and CMIP5 Climate and Hydrology Projections: Release of Downscaled CMIP5 Climate Projections, Comparison with preceding Information, and Summary of User Needs.

[CR66] Reinmann AB, Hutyra LR (2017). Edge effects enhance carbon uptake and its vulnerability to climate change in temperate broadleaf forests. Proc Natl Acad Sci.

[CR67] Scheller RM, Mladenoff DJ (2008). Simulated effects of climate change, fragmentation, and inter-specific competition on tree species migration in northern Wisconsin, USA. Climate Res.

[CR68] Silva LCR, Anand M (2013). Probing for the influence of atmospheric CO2 and climate change on forest ecosystems across biomes. Glob Ecol Biogeogr.

[CR69] Stahle DW, Cook ER, Burnette DJ, Torbenson MCA, Howard IM, Griffin D, Diaz JV, Cook BI, Williams AP, Watson E, Sauchyn DJ, Pederson N, Woodhouse CA, Pederson GT, Meko D, Coulthard B, Crawford CJ (2020). Dynamics, variability, and change in seasonal precipitation reconstructions for North America. J Climate.

[CR70] Suess HE (1955). Radiocarbon concentration in modern wood. Science.

[CR71] Szejner P, Wright WE, Belmecheri S, Meko D, Leavitt SW, Ehleringer JR, Monson RK (2018). Disentangling seasonal and interannual legacies from inferred patterns of forest water and carbon cycling using tree-ring stable isotopes. Glob Change Biol.

[CR72] Tognetti R, Lombardi F, Lasserre B, Cherubini P, Marchetti M (2014). Tree-ring stable isotopes reveal twentieth-century increases in water-use efficiency of *Fagus sylvatica* and *Nothofagus* spp. in Italian and Chilean mountains. PLoS ONE.

[CR73] Vadeboncoeur MA, Jennings KA, Ouimette AP, Asbjornsen H (2020). Correcting tree-ring δ13C time series for tree-size effects in eight temperate tree species. Tree Physiol.

[CR74] van der Sleen P, Groenendijk P, Vlam M, Anten NPR, Boom A, Bongers F, Pons TL, Terburg G, Zuidema PA (2015). No growth stimulation of tropical trees by 150 years of CO2 fertilization but water-use efficiency increased. Nature Geosci.

[CR75] Voelker SL, Muzika R-M, Guyette RP, Stambaugh MC (2006). Historical CO2 growth enhancement declines with age in quercus and pinus. Ecol Monogr.

[CR76] Williams AP, Cook ER, Smerdon JE, Cook BI, Abatzoglou JT, Bolles K, Baek SH, Badger AM, Livneh B (2020). Large contribution from anthropogenic warming to an emerging North American megadrought. Science.

[CR77] Wullschleger SD, Norby RJ, Hendrix DL (1992). Carbon exchange rates, chlorophyll content, and carbohydrate status of two forest tree species exposed to carbon dioxide enrichment. Tree Physiol.

[CR78] Wyckoff PH, Bowers R (2010). Response of the prairie–forest border to climate change: impacts of increasing drought may be mitigated by increasing CO2. J Ecol.

[CR79] Zhou S, Duursma RA, Medlyn BE, Kelly JWG, Prentice IC (2013). How should we model plant responses to drought? An analysis of stomatal and non-stomatal responses to water stress. Agric For Meteorol.

[CR80] Zweifel R, Sterck F (2018). A conceptual tree model explaining legacy effects on stem growth. Front For Glob Change.

