# Supplementary material for: Increased water use efficiency leads to decreased precipitation sensitivity of tree growth, but is offset by high temperatures
Source: Oecologia. 2021 Mar 20;197(4):1095–110. doi: 10.1007/s00442-021-04892-0 (PMC8591026; doi:10.1007/s00442-021-04892-0)
Supplement: Supplementary file 1 — Supplementary file1 (PDF 6279 KB) [file 442_2021_4892_MOESM1_ESM.pdf]

Supplemental Tables:

Table S1: Site level tree growth summary statistics

Site #	Site	Structure	Mean Age	Time span	Intercorrelation	AR	EPS	Rbar
1	AVO	Forest	76	1901 - 2015	0.602	0.602	0.924	0.519
2	BON	Savanna	116	1818 - 2015	0.557	0.557	0.871	0.237
3	ENG	Forest	68	1934 - 2015	0.524	0.524	0.850	0.299
4	GLA	Savanna	125	1851 - 2015	0.575	0.575	0.919	0.342
5	GLL1	Forest	105	1877 - 2016	0.536	0.536	0.863	0.268
6	GLL2	Savanna	83	1894 - 2016	0.623	0.623	0.943	0.565
7	GLL3	Savanna	73	1893 - 2016	0.630	0.630	0.905	0.474
8	MOU	Forest	76	1901 - 2015	0.602	0.602	0.924	0.519
9	UNC	Savanna	52	1889 - 2016	0.435	0.435	0.754	0.168

Table S2: Site and age class cohort summaries of climate and growth.

Site #	Site	Structure	Cohort	Trees	Average June Tmax	Average Water Year Precipitation (mm)	Diameter	Mean Ring Width (mm)	Average Interseries Correlation
1	AVO	Forest	Modern	3	24.25	747.93	27.58	2.74	0.61
	AVO	Forest	Past	3	23.56	712.99	13.87	2.23	0.44
2	BON	Savanna	Modern	2	25.19	637.56	31.54	2.43	0.35
	BON	Savanna	Past	7	24.61	592.15	25.52	1.29	0.63
3	ENG	Forest	Modern	2	25.18	715.71	33.95	3.23	0.51
	ENG	Forest	Past	4	24.63	661.04	12.95	2.46	0.51
4	GLA	Savanna	Modern	1	26.86	916.82	45.15	2.44	0.75
	GLA	Savanna	Past	9	26.63	841.66	31.40	1.94	0.62
5	GLL1	Forest	Modern	1	25.19	637.56	55.43	1.29	0.39
	GLL1	Forest	Past	12	24.61	592.15	17.23	1.46	0.55
6	GLL2	Savanna	Modern	8	25.19	637.56	23.70	1.40	0.62
	GLL2	Savanna	Past	4	24.61	592.15	22.13	1.45	0.58
7	GLL3	Savanna	Modern	2	25.19	637.56	9.95	1.00	0.51
	GLL3	Savanna	Past	7	24.61	592.15	17.72	1.57	0.50
8	MOU	Forest	Modern	2	25.45	829.90	33.06	4.71	0.30
	MOU	Forest	Past	4	25.25	766.07	32.38	2.54	0.49
9	UNC	Savanna	Modern	28	24.25	747.93	13.80	1.53	0.48
	UNC	Savanna	Past	4	23.86	689.23	20.34	1.12	0.48

Table S3: Model parameter estimates and model selection

Parameter	Cohort	Model 1 Estimate	Model 1 95% CI	Model 2 Structure	Model 2 Estimate	95% CI
Intercept	UNC	0.066 *	(0 - 0.13)	UNC	0.067	(0 - 0.13)
	MOU	0.131 *	(0.03 - 0.24)	MOU	0.222 *	(0.08 - 0.37)
	GLL3	0.142 *	(0.04 - 0.24)	GLL3	0.149 *	(0.05 - 0.25)
	GLL2	0.076	(-0.01 - 0.16)	GLL2	0.096 *	(0.01 - 0.18)
	GLL1	0.055	(-0.03 - 0.13)	GLL1	0.035	(-0.09 - 0.16)
	GLA	0.148 *	(0.07 - 0.23)	GLA	0.139 *	(0.06 - 0.23)
	ENG	0.15 *	(0.04 - 0.27)	ENG	0.177 *	(0.05 - 0.32)
	BON	0.133 *	(0.04 - 0.23)	BON	0.14 *	(0.04 - 0.24)
	AVO	0.088	(-0.02 - 0.19)	AVO	0.121	(-0.01 - 0.25)
DBH	Past	0.044	(-0.01 - 0.09)	Past-Forest	0.013	(-0.09 - 0.1)
				Past-Savanna	0.046	(-0.01 - 0.1)
	Modern	0.016	(-0.02 - 0.05)	Modern-Forest	0.035	(-0.05 - 0.11)
				Modern-Savanna	0	(-0.05 - 0.04)
Lag-1	Past	0.56 *	(0.48 - 0.64)	Past-Forest	0.513 *	(0.41 - 0.62)
				Past-Savanna	0.578 *	(0.48 - 0.68)
	Modern	0.482 *	(0.37 - 0.58)	Modern-Forest	0.55 *	(0.37 - 0.74)
				Modern-Savanna	0.489 *	(0.37 - 0.59)
Lag-2	Past	0.197 *	(0.12 - 0.27)	Past-Forest	0.156 *	(0.05 - 0.27)
				Past-Savanna	0.216 *	(0.12 - 0.31)
	Modern	0.375 *	(0.27 - 0.48)	Modern-Forest	0.232 *	(0.04 - 0.4)
				Modern-Savanna	0.387 *	(0.29 - 0.51)
Precipitation	Past	0.112 *	(0.06 - 0.16)	Past-Forest	0.129 *	(0.02 - 0.24)
				Past-Savanna	0.111 *	(0.06 - 0.17)
	Modern	0.017	(-0.05 - 0.08)	Modern-Forest	0.029	(-0.08 - 0.14)
				Modern-Savanna	0.014	(-0.06 - 0.09)
June Tmax x Precip	Past	-0.021	(-0.05 - 0.01)	Past-Forest	-0.058	(-0.13 - 0.02)
				Past-Savanna	-0.012	(-0.05 - 0.02)
	Modern	0.034	(-0.01 - 0.08)	Modern-Forest	-0.021	(-0.1 - 0.06)
				Modern-Savanna	0.064 *	(0.01 - 0.12)
June Maximum Temperature	Past	-0.072 *	(-0.12 - -0.03)	Past-Forest	-0.094 *	(-0.19 - -0.01)
				Past-Savanna	-0.073 *	(-0.13 - -0.02)
	Modern	-0.076 *	(-0.14 - -0.01)	Modern-Forest	-0.081	(-0.18 - 0.03)
				Modern-Savanna	-0.054	(-0.12 - 0.01)
**Model Fit**						
	modelname	Model 1			Model 2	
	Mean Sq. Error	0.235			1.400	
	Bias	0.005			-0.027	
	R sq	0.729			0.739	
	penalties	19.722			29.322	
	deviances	499.295			486.124	
	penalized deviance	519.017			515.446	

* 95% CI does not contain zero

Table S4: Site and samples selected for $\delta^{13}C$ and iWUE analysis and mean estimates of $\delta^{13}C$ (corrected for the Suess effect) and iWUE

Site Number	Site	Cohort	Structure	Number of Trees	Number of Years	$\delta^{13}C$	Average iWUE
1	GLA	Modern	Savanna	3	18	-24.27	138.09
	GLA	Past	Savanna	2	11	-25.15	128.88
2	GLL2	Modern	Savanna	5	47	-24.73	144.57
	GLL2	Past	Savanna	1	21	-23.97	119.37
3	MOU	Modern	Forest	3	11	-23.98	138.59
	MOU	Past	Forest	2	14	-23.27	114.64
4	UNC	Modern	Savanna	2	27	-24.19	139.48
	UNC	Past	Savanna	1	17	-25.01	128.15
5	BON	Modern	Savanna	3	32	-24.64	150.68
	BON	Past	Savanna	2	48	-25.27	129.95

Table S5: Cohort-only δ^{13}C and iWUE model parameter estimates

Parameter	Cohort	δ 13C model		WUE model	
		Estimate	95% CI	Estimate	95% CI
Baseline intercept	Past	-24.218 *	(-24.48 - -23.94)	122.879 *	(119.39 - 126.55)
	Modern	-24.622 *	(-24.76 - -24.48)	144.228 *	(142.39 - 146.18)
Precip	Past	0.352 *	(0.13 - 0.56)	-3.232 *	(-6.17 - -0.06)
	Modern	-0.122	(-0.25 - 0.01)	1.172	(-0.63 - 2.96)
June Tmax	Past	0.13	(-0.03 - 0.28)	-1.821	(-3.63 - 0.13)
	Modern	0.127 *	(0 - 0.26)	-2.457 *	(-4.02 - -0.92)
DBH	Past	0.267	(-0.02 - 0.57)	0.302	(-4.37 - 3.24)
	Modern	0.084	(-0.04 - 0.21)	2.278 *	(0.66 - 3.98)

* 95% CI does not contain zero

Table S6: Cohort and structure $\delta^{13}C$ and iWUE model estimates

Parameter	Cohort	δ 13C model		WUE model	
		Estimate	95% CI	Estimate	95% CI
Baseline Intercept	Past-Savanna	-24.894 *	(-25.23 - -24.55)	128.207 *	(123.34 - 132.81)
	Past-Forest	-23.119 *	(-23.53 - -22.68)	113.508 *	(107.46 - 119.44)
	Modern-Savanna	-24.66 *	(-24.79 - -24.53)	144.528 *	(142.63 - 146.42)
	Modern-Forest	-24.107 *	(-24.69 - -23.52)	137.423 *	(130.16 - 145.4)
Precip	Past-Savanna	0.105	(-0.11 - 0.34)	-0.706	(-4.1 - 1.86)
	Past-Forest	-0.108	(-0.51 - 0.28)	0.592	(-3.72 - 4.65)
	Modern-Savanna	-0.123 *	(-0.24 - -0.01)	1.044	(-0.53 - 2.68)
	Modern-Forest	-0.317	(-0.79 - 0.07)	2.52	(-1.13 - 9.64)
June Tmax	Past-Savanna	-0.004	(-0.17 - 0.16)	-0.775	(-2.77 - 1.67)
	Past-Forest	0.09	(-0.18 - 0.36)	-1.376	(-3.95 - 1.79)
	Modern-Savanna	0.128 *	(0.01 - 0.26)	-2.47 *	(-4.29 - -0.79)
	Modern-Forest	0.075	(-0.25 - 0.43)	-1.318	(-4.68 - 3.07)
DBH	Past-Savanna	0.053	(-0.26 - 0.36)	1.164	(-2.81 - 4.18)
	Past-Forest	0.032	(-0.71 - 0.68)	1.7	(-4.85 - 7.97)
	Modern-Savanna	0.015	(-0.11 - 0.13)	2.976 *	(1.28 - 4.78)
	Modern-Forest	0.597 *	(0.08 - 1.12)	-0.531	(-7.54 - 3.71)

* 95% CI does not contain zero

Table S7: Ensemble of downscaled climate model projections and representative concentration pathways used to determine the range of future June maximum temperatures.

model	rcp26	rcp45	rcp60	rcp85
access1-0.1		x		x
bcc-csm1-1-m.1		x		x
bcc-csm1-1.1	x	x	x	x
canesm2.1	x	x		x
canesm2.2	x	x		x
canesm2.3	x	x		x
canesm2.4	x	x		x
canesm2.5	x	x		x
ccsm4.1	x	x	x	x
ccsm4.2	x	x	x	x
ccsm4.3	x	x	x	x
ccsm4.4	x	x	x	x
ccsm4.5	x	x	x	x
cesm1-bgc.1		x		x
cesm1-cam5.1	x	x	x	x
cesm1-cam5.2	x	x		x
cesm1-cam5.3	x	x	x	x
cmcc-cm.1		x		x
cnrm-cm5.1		x		x
cnrm-cm5.10				x
cnrm-cm5.2				x
cnrm-cm5.4				x
cnrm-cm5.6				x
csiro-mk3-6-0.1	x	x	x	x
ec-earth.12	x	x		x
ec-earth.2		x		
ec-earth.6				x
ec-earth.8	x	x		x
fgoals-g2.1	x	x		x
fio-esm.1	x	x	x	x
fio-esm.2	x	x	x	x
fio-esm.3	x	x	x	x
gfdl-cm3.1	x	x	x	x
gfdl-esm2g.1	x	x	x	x
gfdl-esm2m.1	x	x	x	x
giss-e2-h-cc.1		x		
giss-e2-r-cc.1		x		
giss-e2-r.1	x	x	x	x
giss-e2-r.2		x		
giss-e2-r.3		x		
giss-e2-r.4		x		
giss-e2-r.5		x		
hadgem2-ao.1	x	x	x	x
hadgem2-cc.1		x		x
hadgem2-es.1	x	x	x	x
hadgem2-es.2	x	x	x	x
hadgem2-es.3	x	x	x	x
hadgem2-es.4	x	x	x	x
inmcm4.1		x		x
ipsl-cm5a-lr.1	x	x	x	x
ipsl-cm5a-lr.2	x	x		x
ipsl-cm5a-lr.3	x	x		x
ipsl-cm5a-lr.4		x		x
ipsl-cm5a-mr.1	x	x	x	x
ipsl-cm5b-lr.1		x		x
miroc-esm-chem.1	x	x	x	x
miroc-esm.1	x	x	x	x
miroc5.1	x	x	x	x
mpi-esm-lr.1	x	x		x
mpi-esm-lr.2	x	x		x
mpi-esm-lr.3	x	x		x
mpi-esm-mr.1	x	x		x
mri-cgcm3.1	x	x		

Table S8: Table of the number of trees from each species included in our study.

Table 1: Species included in our analysis

Structure	*Species*	# of trees
Forest	*Quercus macrocarpa*	15
	Quercus rubra	12
	Quercus velutina	2
Savanna	*Quercus alba*	6
	Quercus macrocarpa	53
	Quercus rubra	12

Supplemental figures:

Figure S1: Bootstrapped correlation coefficients between site chronologies and climate variables used to choose the most influential climate variables to include in the Bayesian tree growth mode. A) Tree ring growth correlations with monthly and water year precipitation. B). Tree ring growth correlations with maximum monthly temperatures, C) Tree ring growth correlations with maximum Vapor Pressure Deficit. Bars indicate the mean bootstrapped coefficient by site. Sites are colored by total precipitation (highest = blue, lowest = red).

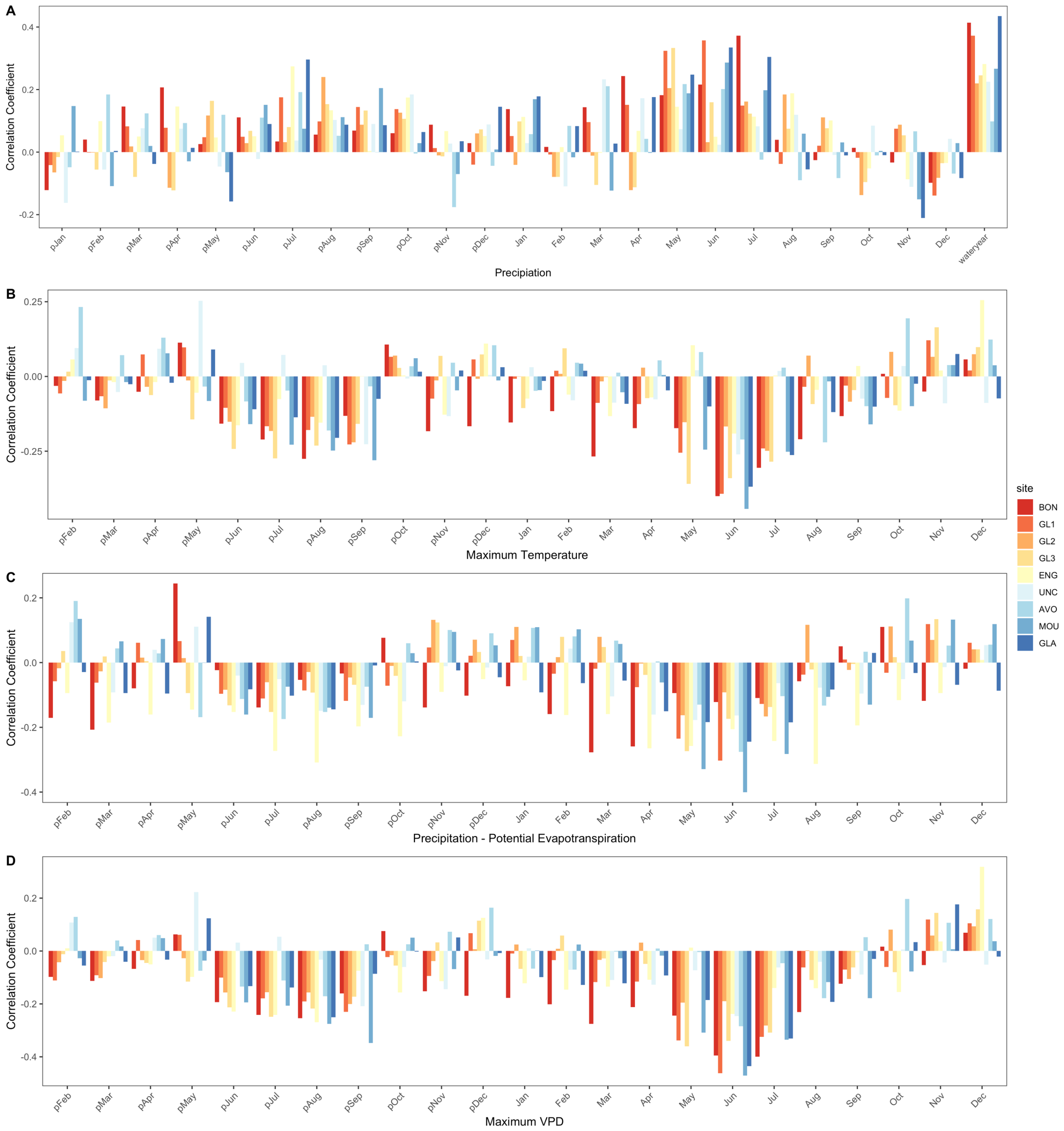

A
Correlation Coefficient
Precipiation
pJan pFeb pMar pApr pMay pJun pJul pAug pSep pOct pNov pDec Jan Feb Mar Apr May Jun Jul Aug Sep Oct Nov Dec wateryear
B
Correlation Coefficient
Maximum Temperature
C
Correlation Coefficient
Precipitation - Potential Evapotranspiration
D
Correlation Coefficient
Maximum VPD
pFeb pMar pApr pMay pJun pJul pAug pSep pOct pNov pDec Jan Feb Mar Apr May Jun Jul Aug Sep Oct Nov Dec
site
BON
GL1
GL2
GL3
ENG
UNC
AVO
MOU
GLA

Figure S2: Moving correlation plots of site level chronologies with site level climate parameters show consistent trends with model estimated parameters presented here. A). Most sites show declining sensitivity to annual water year precipitation over time. B). At the same time, many sites show increasing correlations with summer maximum temperatures, particularly from 1945-2005. Site chronologies correlations presented here were detrended using splines to remove long term trends. Grey shading indicates time periods where we did not at least 5 time series to generate a site chronology.

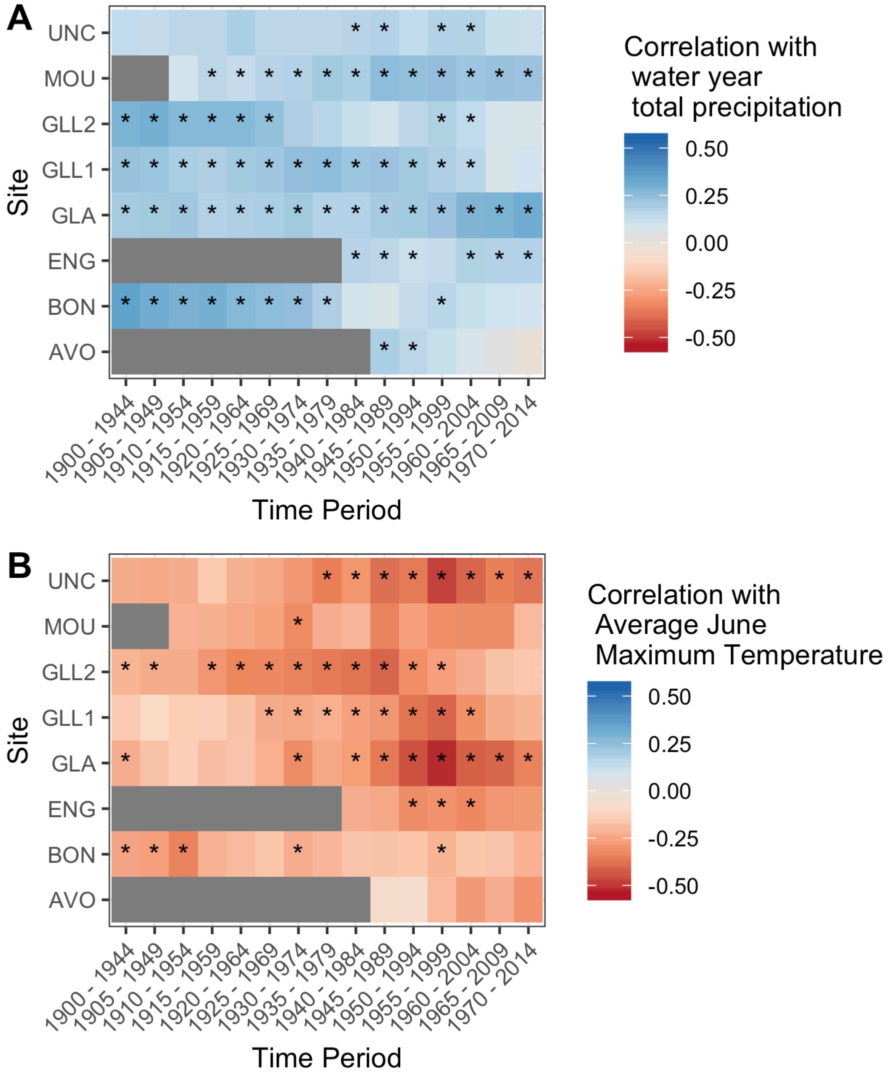

Figure S3: Preliminary analysis between site-specific linear regressions between spline detrended ring width indices and total annual precipitation (centered and scaled). Red points represent all years after 1950, and blue represents all years before 1950. Many sites indicate a shift in regression slopes between the two periods.

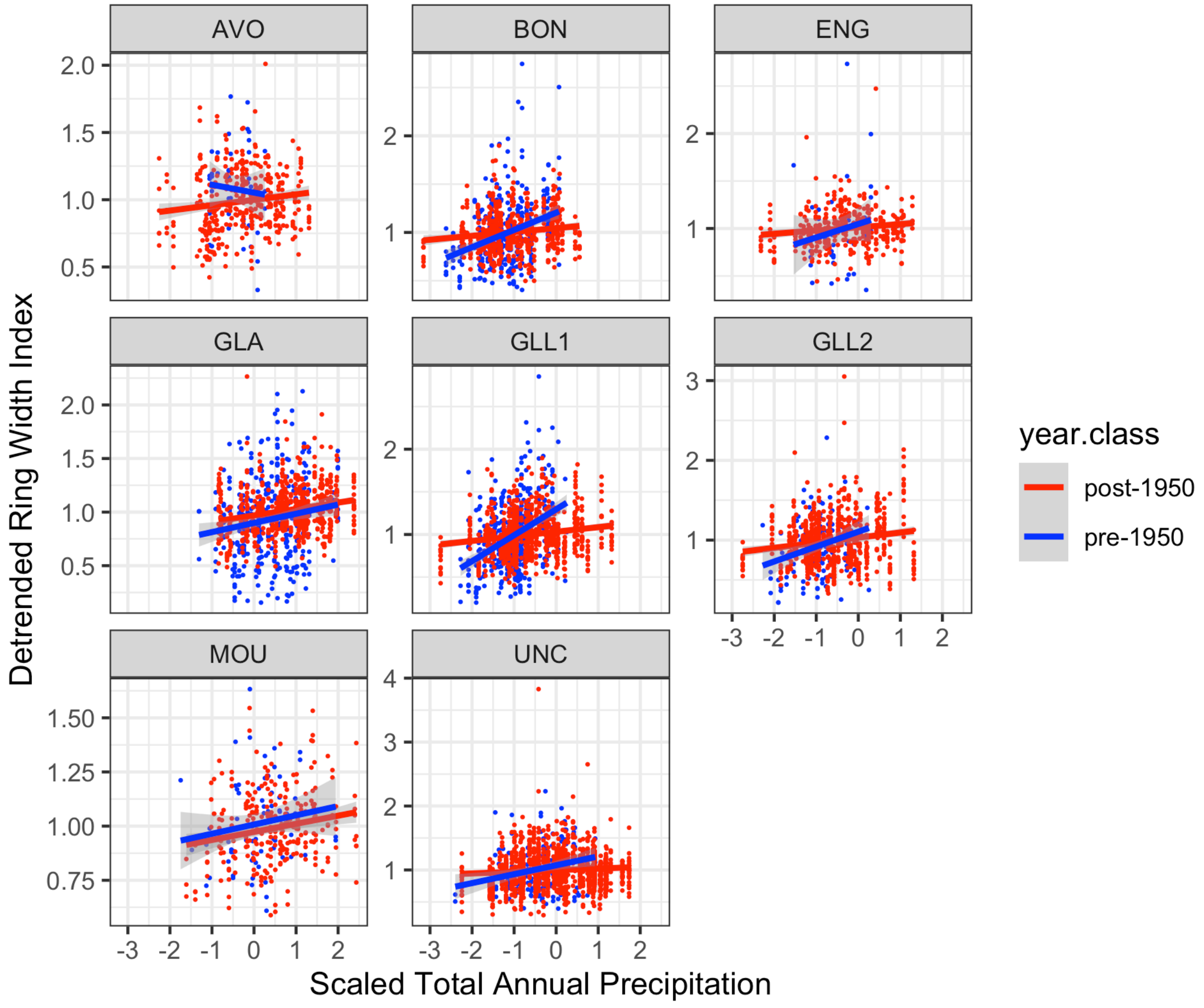

Figure S4: Preliminary analysis between site-specific linear regressions and spline detrended ring width indices and total annual precipitation (centered and scaled). Red points represent all years after 1950 for trees established after 1900 (the past cohort), and blue represents all years before 1950, for trees established after 1900 (the modern cohort). Many sites indicate a shift in regression slopes between the two periods.

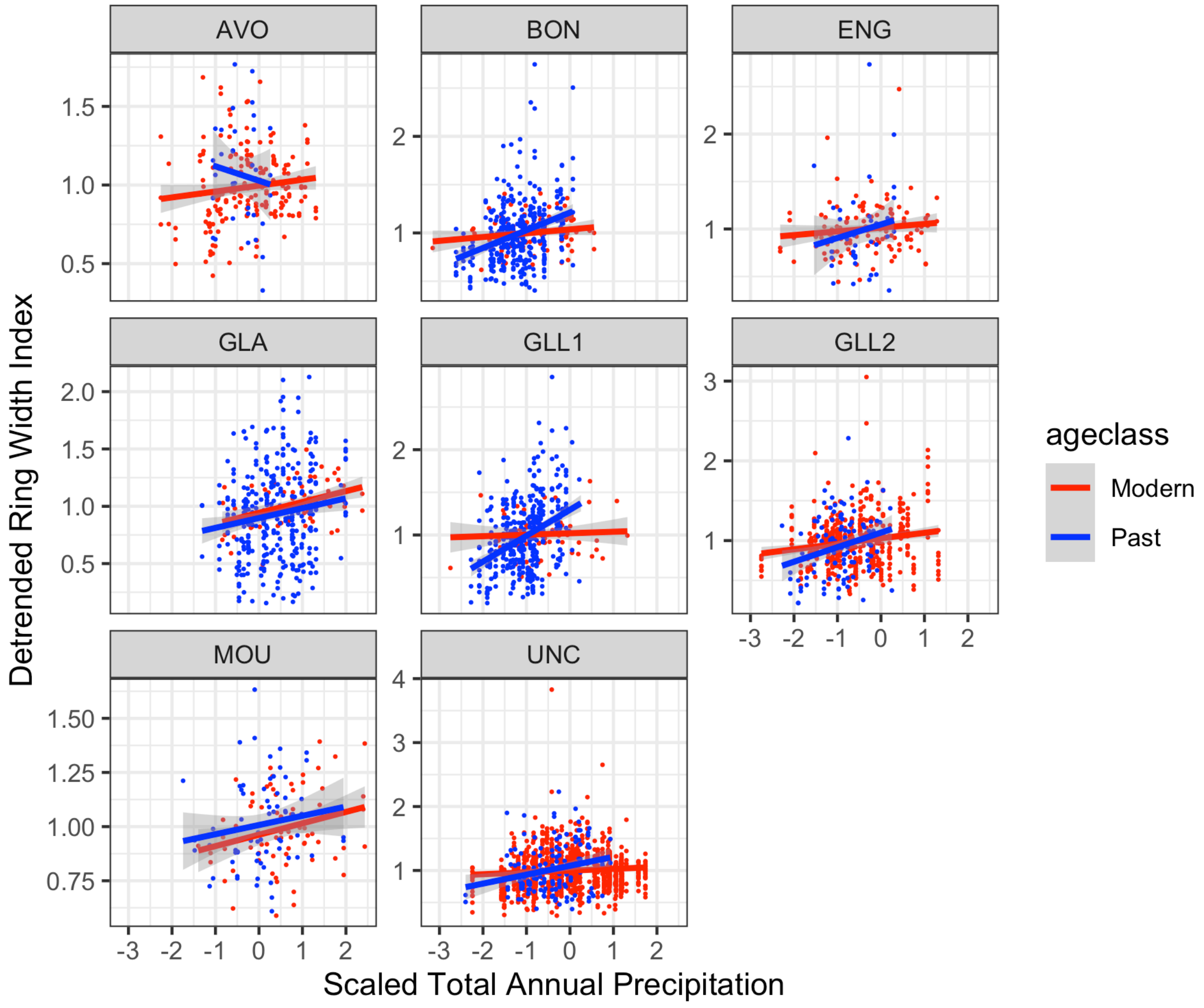

Figure S5: Estimated tree-ring growth model coefficients for models fit with all climate years for the cohort-only model (left column) and the cohort-structure model (right column). Dots indicate mean model coefficient estimates, bars are 95% credible intervals. Compare to Figure 2, which was fit with only the top 25% driest climate years.

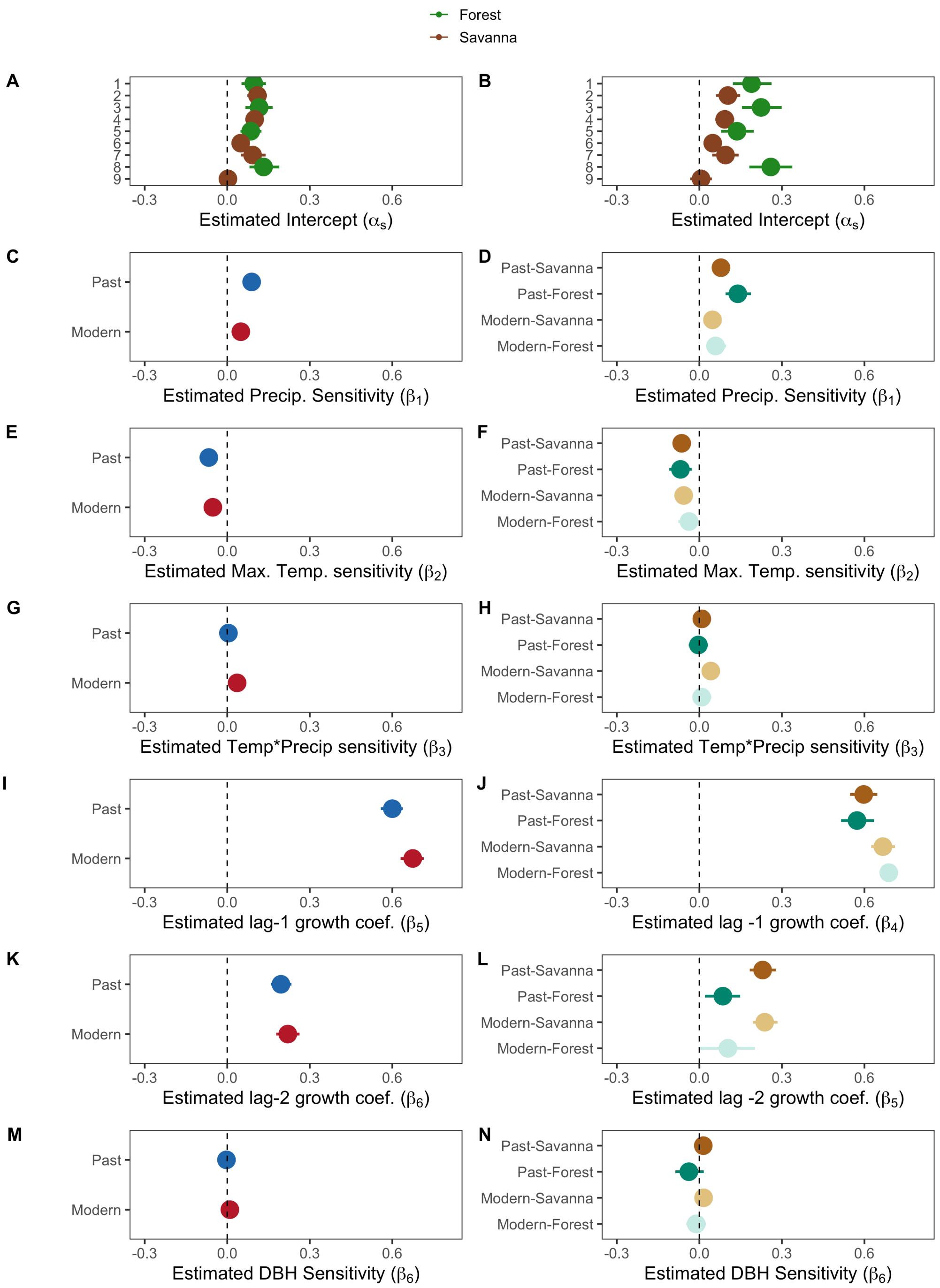

Figure S6: Predicted tree growth vs. observed tree growth for A) selected model with cohort-only random slopes and B) structure-cohort random slopes.

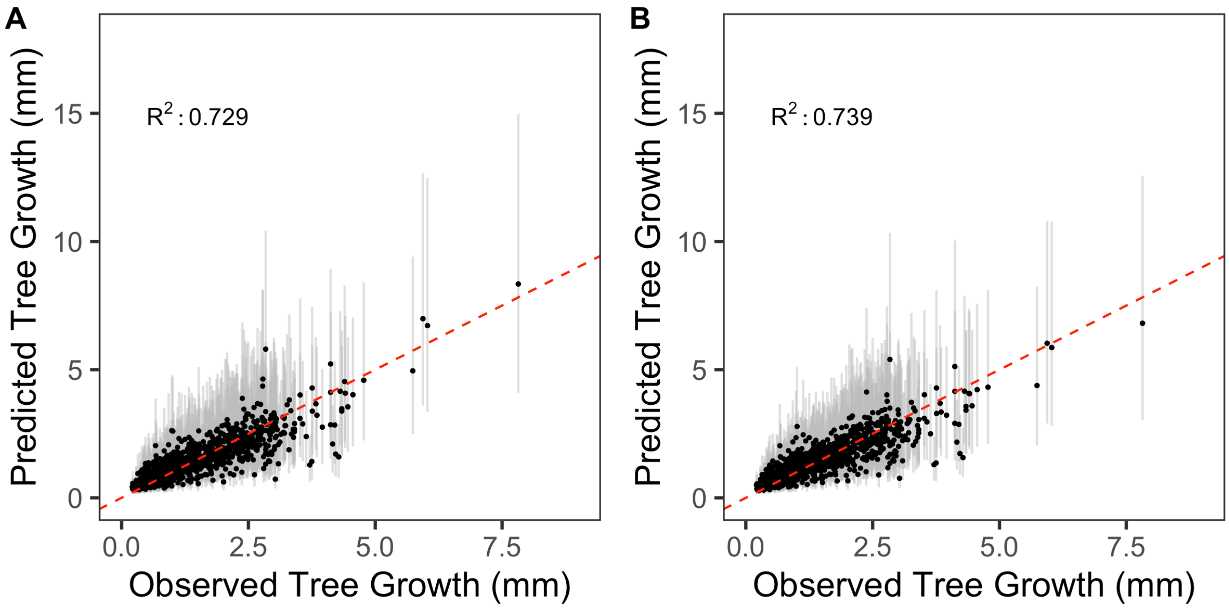

Figure S7: Site-level mean tree ring growth time series predicted from the structure-cohort random slopes model tracks the observed tree ring growth data ($R^2 = 0.73$). Solid lines represent predicted means and red & blue shading are 95% CI for predictions for all modern and past trees (respectively) at each site. Dashed lines represent means of raw data and the yellow and gray shading depict 95% quantiles of tree ring growth for the raw data.

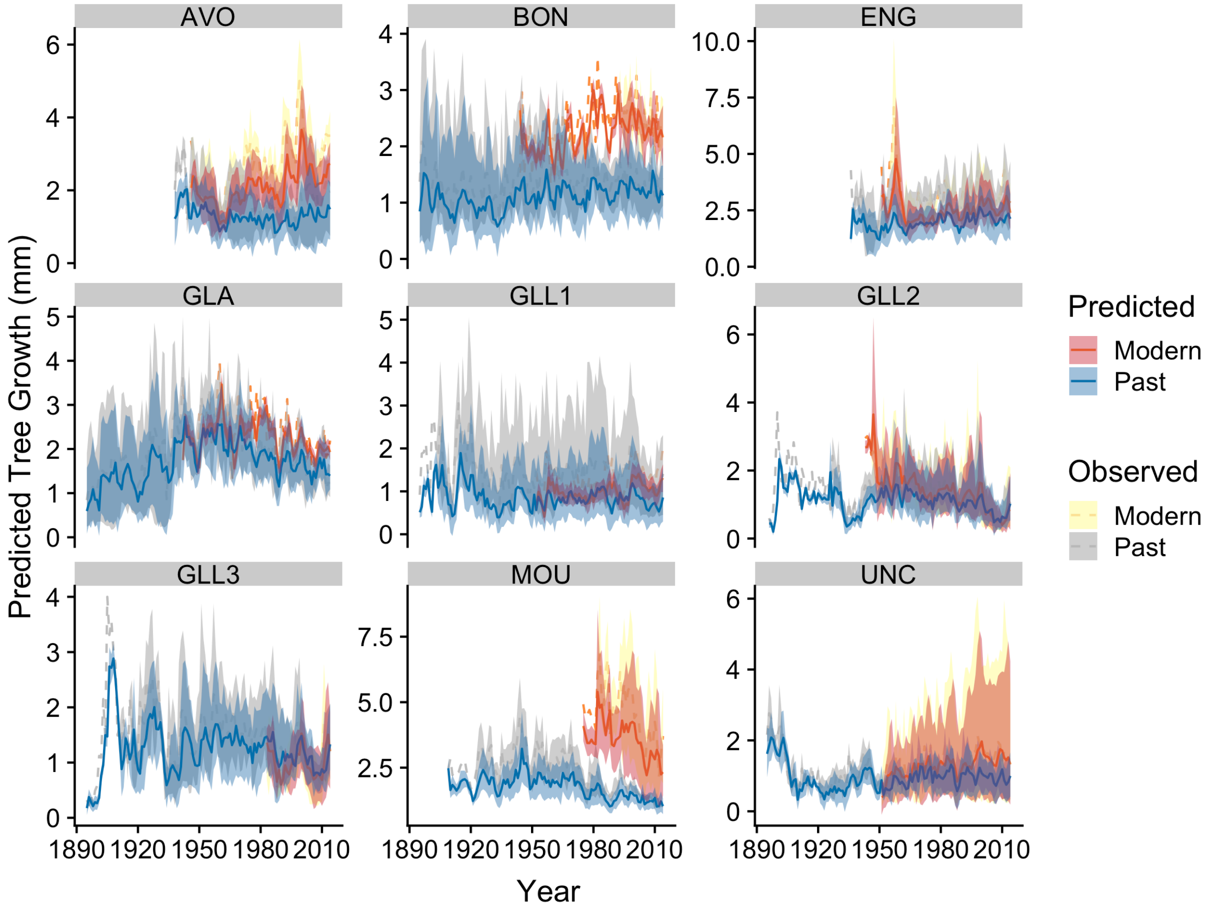

Figure S8: Differences in iWUE, total growth, and growth sensitivity parameters for the cohort-only models. A). Mean predicted iWUE is higher for the modern cohort, but there is B) no change in mean predicted growth across cohorts. C). Shifts in growth model parameter estimates (Modern -Past cohort). Black bars are differences where 95% CI do not overlap with zero.

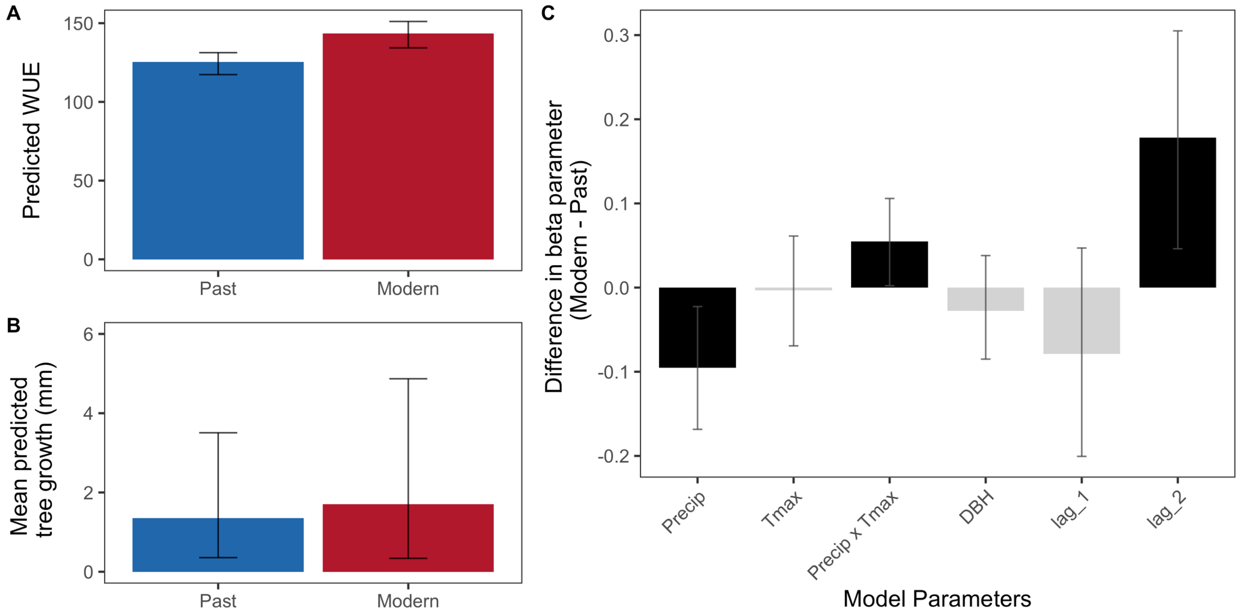

Figure S9: Raw stable isotope time series used in $\delta^{13}C$ and iWUE models. A) $\delta^{13}C$ estimates corrected for Suess effect B) Raw WUE time series

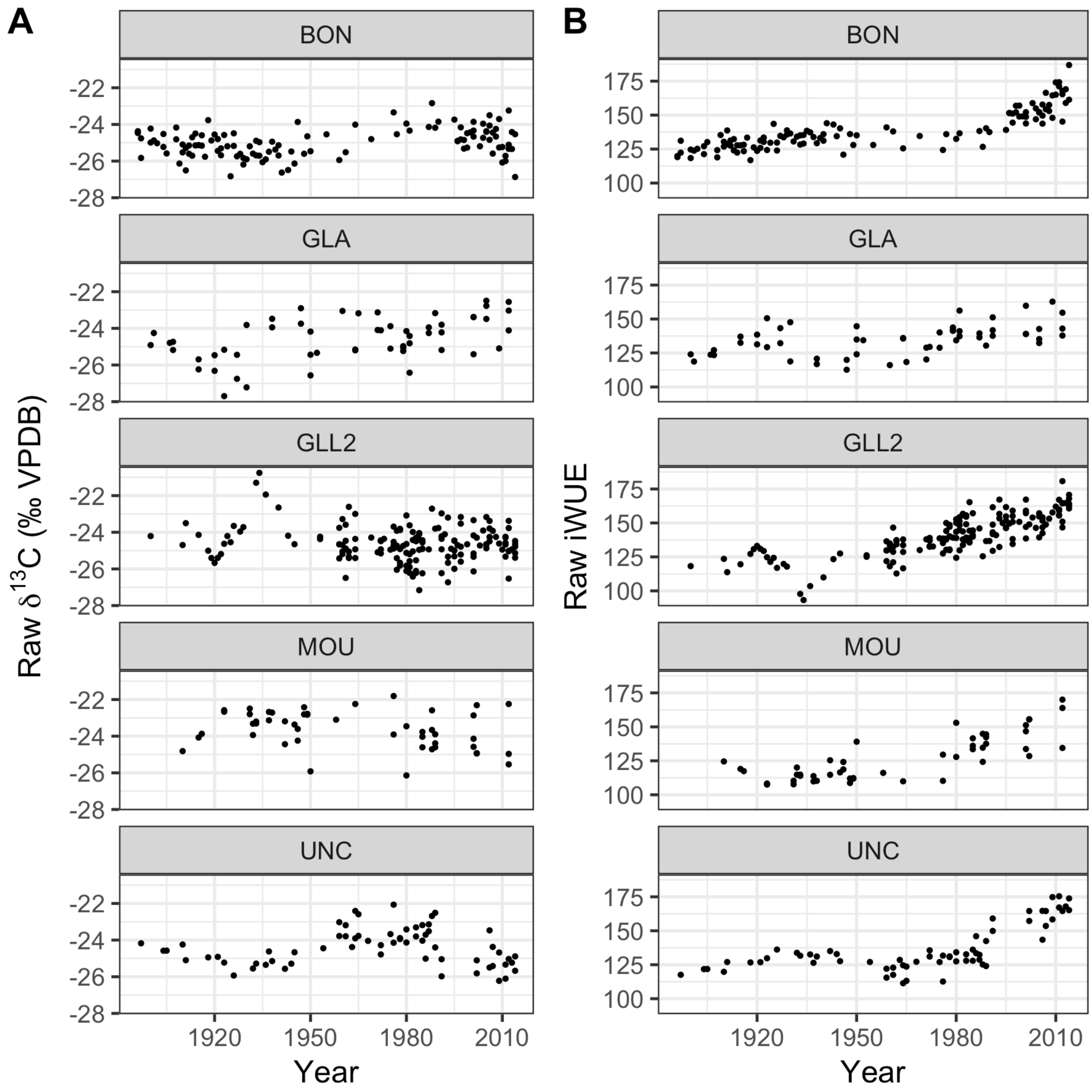

A
B
BON
GLA
GLL2
MOU
UNC
Raw δ13C (‰ VPDB)
Raw iWUE
-22
-24
-26
-28
100
125
150
175
1920
1950
1980
2010
Year

Figure S10: Model predicted versus observed intrinsic WUE for A) age class cohort random slopes model and B) cohort-structure random slopes model.

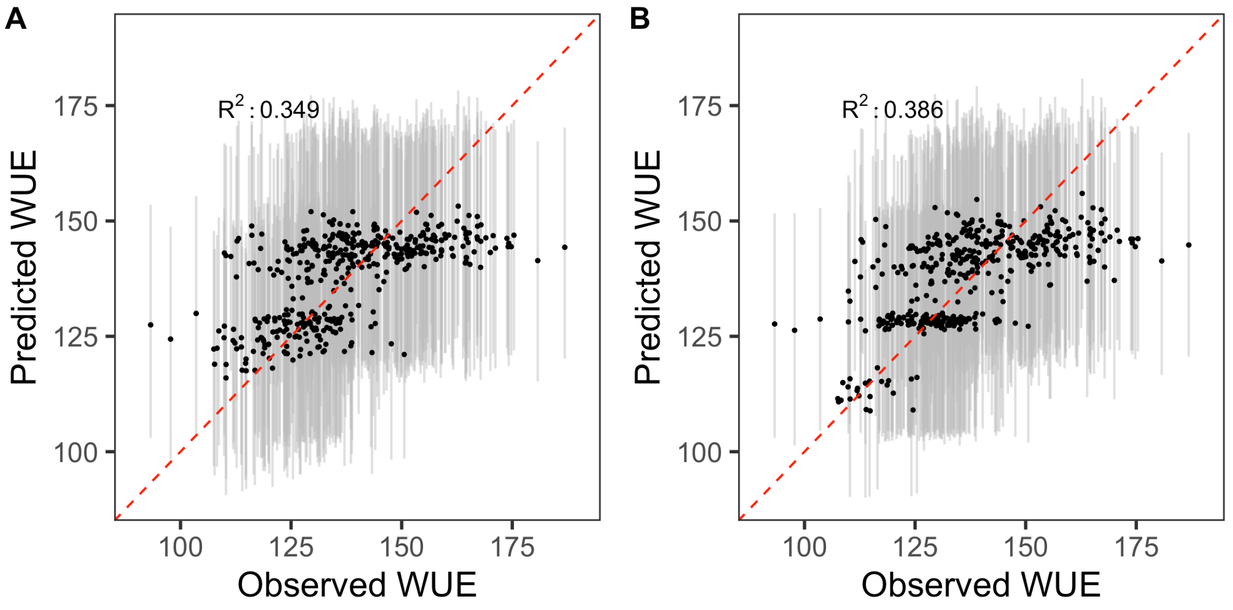

Figure S11: Predicted versus observed $\delta^{13}C$ values (‰) for A) age class cohort random slopes model and B) cohort-structure random slopes model.

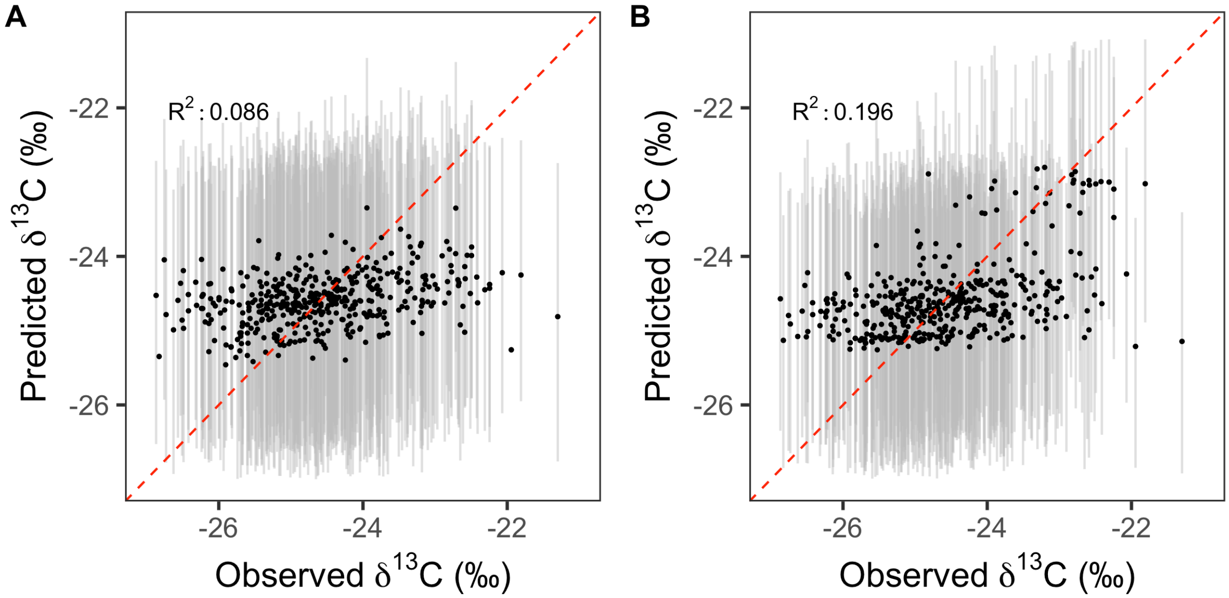

Figure S12: A) Average predicted Water Use Efficiency across stand structure and age classes, and B) Average predicted tree growth across stand structure and age classes.

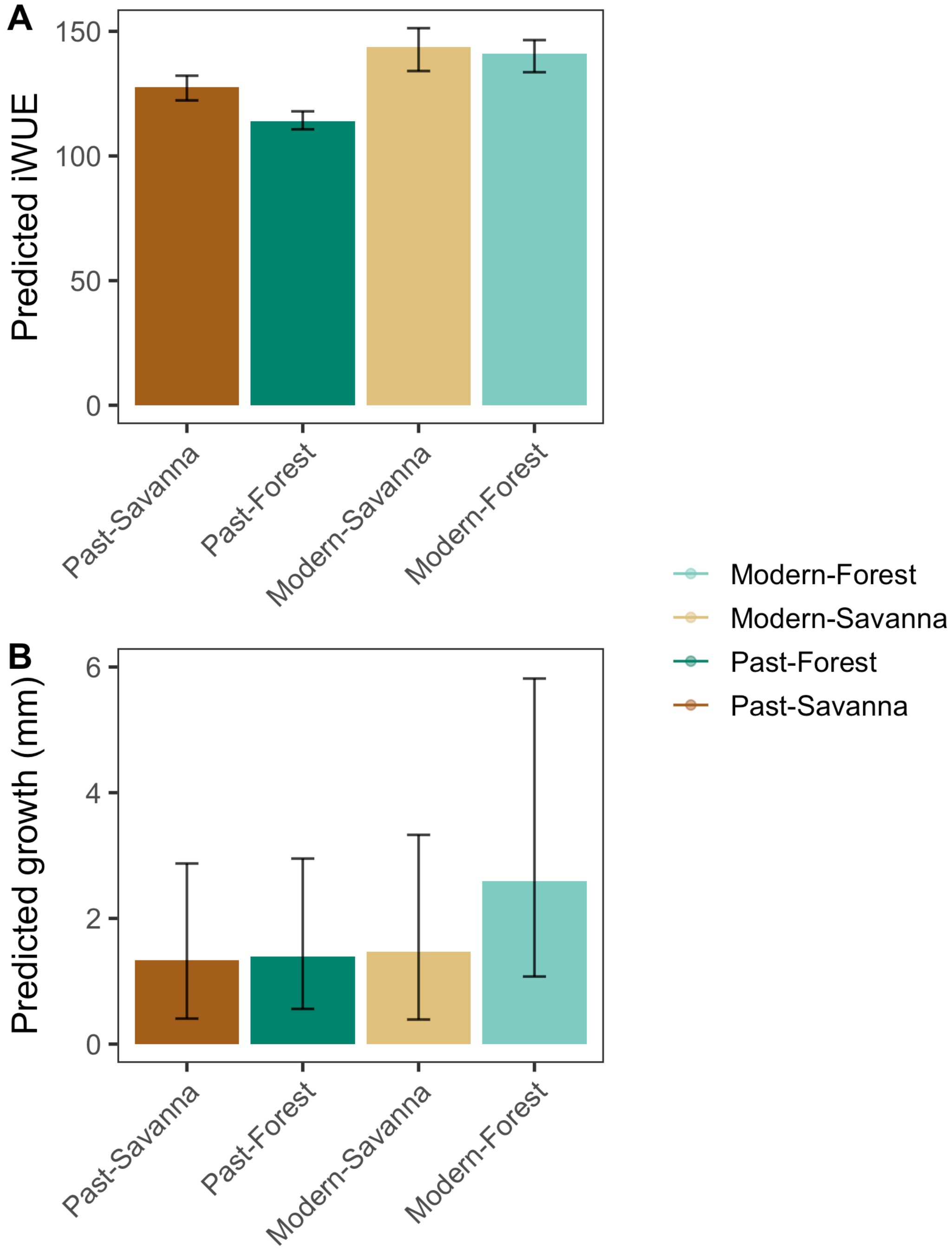

Figure S13: Average change in growth between modern and past cohorts for a tree of average DBH, average previous years' growth, and 25.5°C maximum summer temperature in forest and savanna systems (A), and requisite percent change in growth between modern and the past cohort (B).

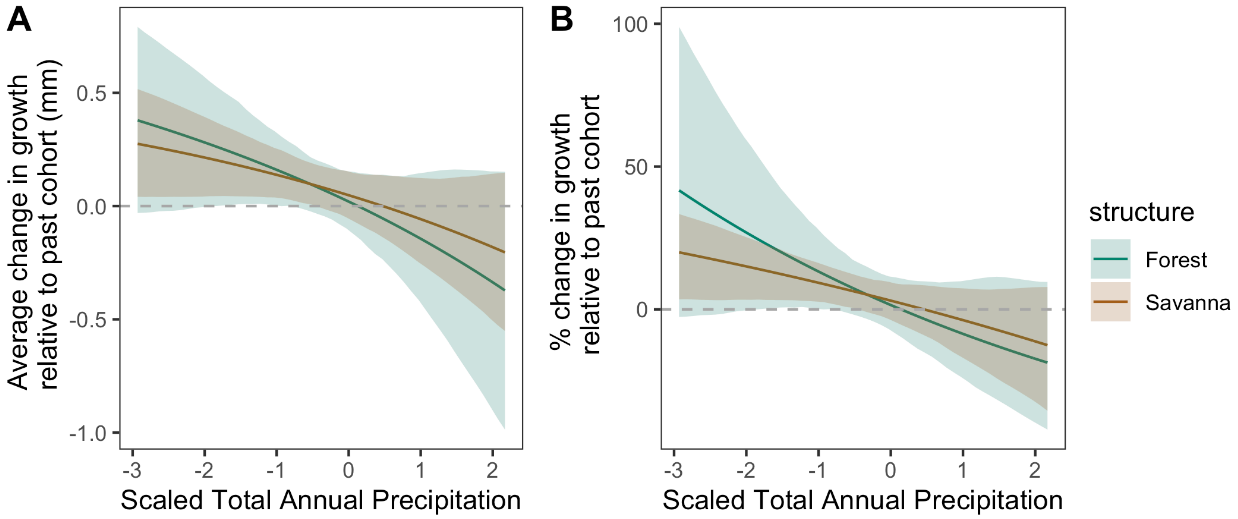

Figure S14: Interaction between temperature and precipitation by cohort and structure classes.

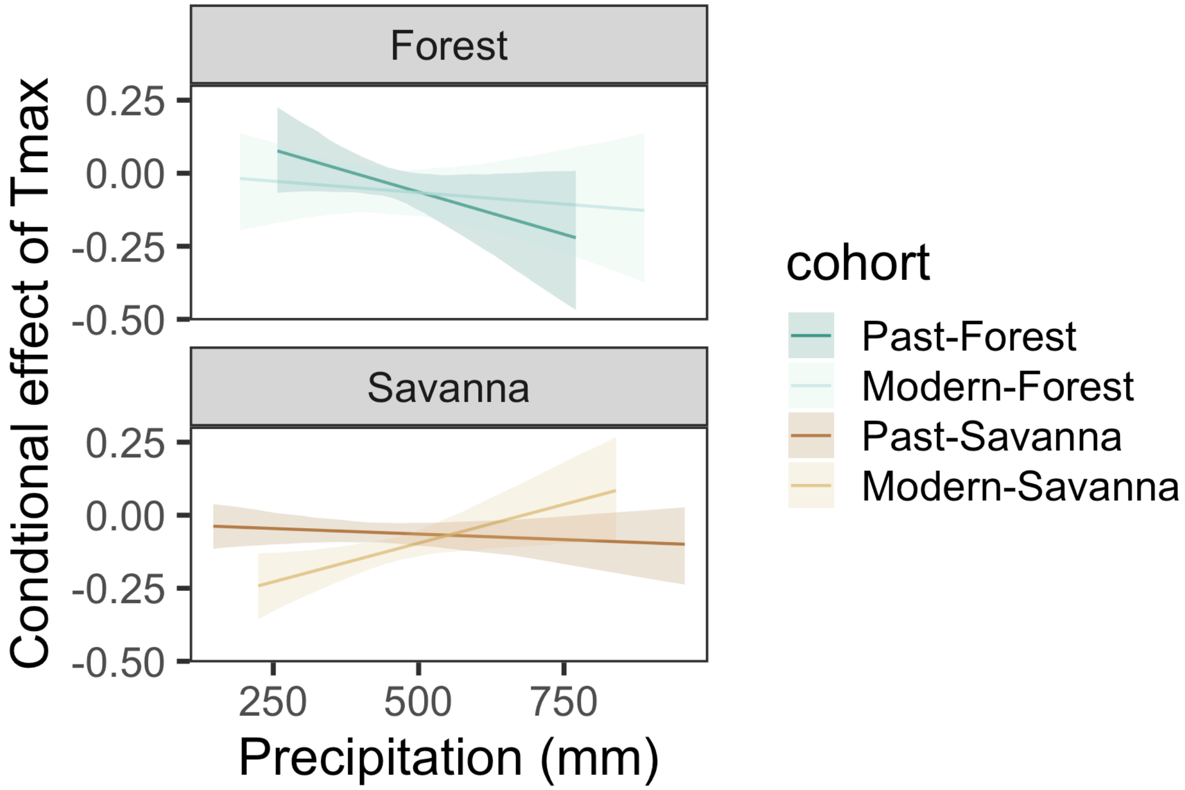

Supplemental JAGS growth model likelihood function and code:
The likelihood function of our model of ring width *(RW)* is as follows, where N is the total number of ring widths modelled, and S is the total number of sites:

$$[\alpha_s, \beta 1 - 6c, mu_{\alpha s,}, mu_{\beta 1-6c}, \sigma^2_{\alpha s}, \sigma^2_{\beta 1-6c}, \sigma^2_p \,|RW]$$
$$\propto \prod_{s=1}^{S} \prod_{i=1}^{N} Normal(\log(RW) \mid g(\alpha, \beta, Precip, DBH, MaxTemp, (growth - 1), (growth - 2), \sigma^2_p)$$
$$x\ normal(\alpha_s\ |mu_\alpha, \sigma^2_\alpha)$$
$$x\ normal(\beta_{1-6c}\ |mu_{\beta 1-6}, \sigma^2_\alpha)$$
$$x\ inverse\ gamma(\sigma^2_{\alpha s}\ |0.001, 0.001)$$
$$x\ inverse\ gamma\left(\sigma^2_{\beta 1-6c}\ \middle|0.001, 0.001\right)$$
$$x\ uniform(mu_\alpha| - 2, 2)$$
$$\text{x}\ uniform\left(mu_\beta\middle| - 2, 2\right)\ inversegamma\left(\sigma^2_p\ \middle|0.001, 0.001\right)$$

JAGS code:

```
Growth.Model <- "model{


# Likelihood

for(i in 1:n){ # n is the number of individual tree-years

# ## ------------Process Model--------------------------
Y[i]   ~ dnorm(gfunc[i], inv.var) # where Yi is already log transformed

# function g()
# site level random intercept (beta1)
# ageclass-structure random slopes for climate (DI.scaled, Temp.scaled + interaction), tree size
(DBH.scaled), and log transformed previous years of growth.

gfunc[i] <- beta1[site[i]] + beta2[age[i]]*DI.scaled[i] +
beta3[age[i]]*DBH.scaled[i]+beta4[age[i]]*log_RWI_1[i]+beta5[age[i]]*log_RWI_2[i]  +
beta6[age[i]]*Temp.scaled[i] + beta7[age[i]]*Temp.scaled[i]*DI.scaled[i]


}

## -------------Priors--------------------
# Assume normal, uninformative priors for betas, but generate a beta + alpha for each ageclass

# cohort random slopes
for(s in 1:2){
```

```
beta2[s] ~ dnorm(mu_beta2, inv_beta2)
beta3[s] ~ dnorm(mu_beta3, inv_beta3)
beta4[s] ~ dnorm(mu_beta4, inv_beta4)
beta5[s] ~ dnorm(mu_beta5, inv_beta5)
beta6[s] ~ dnorm(mu_beta6, inv_beta6)
beta7[s] ~ dnorm(mu_beta7, inv_beta7)
}


# site level random intercept
for(k in 1:Nsites){
beta1[k] ~ dnorm(mu_beta1, inv_beta1)
}


# use uniform priors for each Mu_beta hyperparamter
mu_beta1 ~ dunif(-1, 1)
mu_beta2 ~ dunif(-1, 1)
mu_beta3 ~ dunif(-1, 1)
mu_beta4 ~ dunif(-1, 1)
mu_beta5 ~ dunif(-1, 1)
mu_beta6 ~ dunif(-1, 1)
mu_beta7 ~ dunif(-1, 1)

# priors for error terms
inv_beta1   ~ dgamma(0.01, 0.01)
sigma_beta1 <- 1/sqrt(inv_beta1)
inv_beta2   ~ dgamma(0.01, 0.01)
sigma_beta2 <- 1/sqrt(inv_beta2)
inv_beta3   ~ dgamma(0.01, 0.01)
sigma_beta3 <- 1/sqrt(inv_beta3)
inv_beta4   ~ dgamma(0.01, 0.01)
sigma_beta4 <- 1/sqrt(inv_beta4)
inv_beta5   ~ dgamma(0.01, 0.01)
sigma_beta5 <- 1/sqrt(inv_beta5)
inv_beta6   ~ dgamma(0.01, 0.01)
sigma_beta6 <- 1/sqrt(inv_beta6)
inv_beta7   ~ dgamma(0.01, 0.01)
sigma_beta7 <- 1/sqrt(inv_beta7)


# Non-informative Prior for the inverse additive population variances


inv.var   ~ dgamma(0.001, 0.001)
sigma     <- 1/sqrt(inv.var)
```

}"
